# Risk factors for myocarditis hospitalization and recurrence: a state-wide retrospective observational study

**DOI:** 10.1093/ehjopen/oeaf130

**Published:** 2025-10-09

**Authors:** Timothy Nathan Kwan, Gemma Kwan, David Brieger, Vincent Chow, Leonard Kritharides, Austin Chin Chwan Ng

**Affiliations:** Department of Cardiology, Concord Hospital, The University of Sydney, 1 Hospital Road, Concord, Sydney, NSW 2139, Australia; Department of Immunology, Royal Prince Alfred Hospital, The University of Sydney, 50 Missenden Road, Camperdown, Sydney, NSW 2050, Australia; Department of Cardiology, Concord Hospital, The University of Sydney, 1 Hospital Road, Concord, Sydney, NSW 2139, Australia; Department of Cardiology, Concord Hospital, The University of Sydney, 1 Hospital Road, Concord, Sydney, NSW 2139, Australia; Department of Cardiology, Concord Hospital, The University of Sydney, 1 Hospital Road, Concord, Sydney, NSW 2139, Australia; Department of Cardiology, Concord Hospital, The University of Sydney, 1 Hospital Road, Concord, Sydney, NSW 2139, Australia

**Keywords:** Myocarditis, Heart failure, Epidemiology, COVID-19, Australia

## Abstract

**Aims:**

Myocarditis is a potentially life-threatening condition with diverse aetiologies including viral infections, toxins, and autoimmunity. We aimed to quantify the risk factors of index myocarditis hospitalization and subsequent myocarditis recurrence.

**Methods and results:**

We conducted a retrospective cohort study in New South Wales (NSW), Australia, using the Admitted Patient Data Collection (APDC) of all hospitalized patients. Conditions temporally associated with myocarditis within 30 days of the index admission were identified using conditional logistic regression analysis. In patients with previous myocarditis, risk factors for recurrent myocarditis admission were calculated with both Cox regression using cause-specific hazards and competing risk analysis. There were 4071 cases of index myocarditis from 2004 to 2021. Over a median of 4.8 years of follow-up, there were 124 patients whose myocarditis recurred. Two-thirds of cases were male with an average age of 42 years. Index myocarditis cases were much more common within 30 days of a hospitalization for pericarditis, heart failure, ventricular arrhythmias, COVID-19, and several other cardiac, respiratory, and autoimmune conditions, compared to the baseline risk over the preceding 12 months. Similarly, myocarditis recurrence was more common within 30 days of pericarditis, ventricular arrhythmias, COVID-19, and autoimmune disease. Recurrence was not strongly predicted by any features of the index myocarditis admission. Our analysis is solely based on administrative coding, with limited clinical validation, which introduces potential for misclassification.

**Conclusion:**

In our cohort, myocarditis was more frequently diagnosed following presentations with acute respiratory illness (including COVID-19), autoimmune conditions, or cardiac events including ventricular arrhythmias, atrial fibrillation, and heart failure.

## Graphical Abstract

**Graphical Abstract:**
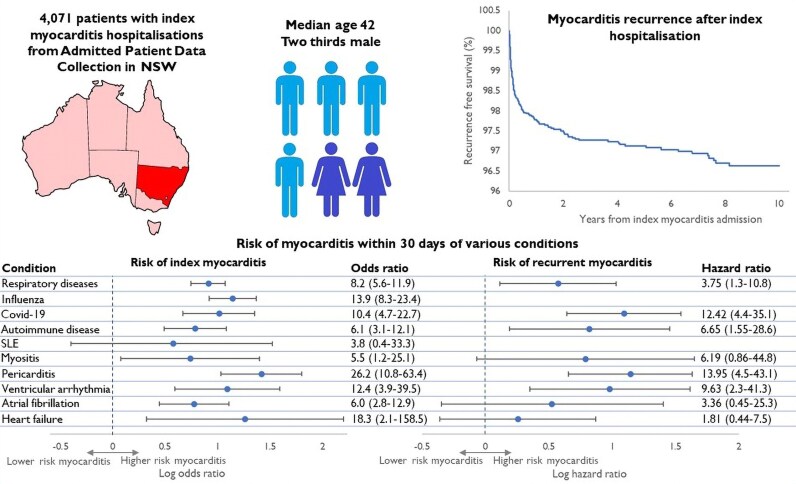

## Introduction

Myocarditis is not common but is a serious condition with rising incidence over the past decade.^1^ The diagnosis of myocarditis is often elusive as it can masquerade as other conditions.^2^ The high burden of undiagnosed myocarditis has been demonstrated in autopsy studies showing the cross-sectional prevalence of myocarditis in general populations is up to 1%.^3–5^ However, patients at risk for myocarditis can be difficult to identify. Due to the rarity of myocarditis and the heterogeneity of this disease, reported data quantifying potential clinical risk factors for myocarditis remains sparse.

There are some recognized and newly identified risk factors for myocarditis. Infections, especially from viruses, are generally seen as the most common cause of myocarditis.^6^ COVID-19 and COVID-19 mRNA vaccination have recently been reported as leading causes of myocarditis.^7^ Immune checkpoint inhibitors and other chemotherapeutics are a major component of a growing category of drug-induced myocarditis emerging during the treatment of malignancy.^8^ Systemic autoimmune diseases, though relatively less common, include myocarditis as a possible disease phenotype.^9^ Demographic risk factors, including male sex, have also been reported as a risk factor for myocarditis.^1^ Most of the reported risk factors have been recognized in an *ad hoc* fashion or involved selected cohorts. There is little data quantifying the real-world associations with myocarditis in a hospitalized cohort at a population level.

We aimed to determine the risk factors of first myocarditis presentation as well as the risk factors for myocarditis readmission following index diagnosis in a state-wide general population.

## Methods

In this population-wide cohort study, data were linked from the New South Wales (NSW, Australia’s most populous state) Admitted Patient Data Collection (APDC) database and the NSW Registry of Births, Deaths and Marriages. This database covers 97% of NSW healthcare facility admission records between 2001 and 2022 inclusive. Data were extracted on patients who had a primary or secondary diagnosis of myocarditis during an admission during this time, excluding the first 3 years and the last 6 months of follow-up to mandate a lookback and follow-up period (see Supplementary material online, *Table S1*). As such, patients were included with admissions ranging from 2004 to 2021. Patients were excluded if they were not NSW residents to maximize follow-up. Disease diagnosis was provided by the International Classification of Diseases Code (see Supplementary material online, *Table S2*). This cohort has been described before.^10^

### Definitions

Recurrent myocarditis was defined as a new admission for myocarditis at least 2 days after discharge from the index myocarditis hospitalization.^11^ The recurrence of myocarditis was required to be the primary diagnosis. Autoimmune and malignant myocarditis were defined as having a background history of autoimmune disease or malignancy, respectively. Reactive myocarditis was defined as having an admission for a respiratory or digestive system condition in the 30 days prior to the myocarditis admission.^10^ Respiratory and digestive system conditions were defined broadly according to any ICD-10 code within that category (see Supplementary material online, *Table S1*).

Geographical location was quantified by the Australian Statistical Geography Standard which does vary from major cities to very remote areas in Australia.^12^ For all analyses, an inpatient spell was defined as a sequence of hospitalizations separated by interhospital transfer and no less than 2 days.^13,14^ When admission duration was reported, this was the duration of the inpatient spell. Similarly, the timing of repeat hospitalizations or myocarditis recurrence was defined relative to this inpatient spell. For example, if a patient had a diagnosis of myocarditis during an index admission and was then transferred to a quaternary centre where this diagnosis of myocarditis remained, this was within a single spell and not a recurrence of myocarditis.

### Statistical analysis

Summary statistics for the study cohort were produced with standard statistical techniques. Survival free of myocarditis recurrence was calculated using the methods of Fine and Gray, where death was the competing event. These techniques have previously been described for the cohort in the present study.^10^ The change in classification from index to recurrent presentation was displayed using a river plot.

All statistical analyses were performed using R 4.3.1.^15,16^ Ethics approval was granted by NSW Population and Health Services Research Ethics Committee, reference number 2019/ETH01790, who also granted a waiver of the usual requirement for the consent of the individual to the use of their health information. The study was conducted in accordance with the Ethical Principles as outlined in the 2024 Declaration of Helsinki.

### Risk factors for index myocarditis

The presence of risk factors prior to myocarditis diagnosis was assessed with conditional logistic regression to compare the frequency of risk factors 1–30 days prior to myocarditis diagnosis to a year prior (the reference group being from 31 to 390 days prior to myocarditis diagnosis). As such each patient provided 1 outcome group and 12 reference groups to generate a single odds ratio for each potential risk factor of interest (see Supplementary material online, *Figure S1*). Multivariable adjustment was not performed as patients acted as their own controls. Prespecified risk factors considered for myocarditis were admissions for COVID-19, influenza, systemic lupus erythematosus, sarcoidosis, myositis, diabetes, malignancy, pericarditis, heart failure, arrhythmia, myocardial infarction, stroke, and broader disease categories: autoimmune disease and digestive and respiratory disease. These risk factors are based on prior research and clinical plausibility.^7,8,9,17^ In an exploratory analysis, common infectious diseases were similarly analysed. All considered risk factors were required to be a primary diagnosis; however, as COVID-19 was a new disease and not coded as a primary diagnosis during the study period within the APDC database, it was identified as a secondary diagnosis.

The frequency of risk factors was plotted, with their frequency also displayed with locally estimated scatterplot smoothing.

Sensitivity analysis was performed whereby the association with possible risk factors 90–120 days prior to myocarditis was compared to 121–480 days. In addition, we applied a third conditional logistic regression model assessing the rates of risk factors 1–90 days prior to myocarditis compared to 91–450 days prior. To determine the robustness and validity of the risk factors association with myocarditis, we replicated our analysis using a positive control cohort of patients admitted for pericarditis and a negative control cohort of patients admitted for hip fracture repair.

### Risk factors for recurrent myocarditis

The risk of myocarditis recurrence was quantified with a Cox regression model with cause-specific hazards. For Cox regression, variables considered as risk factors for recurrent myocarditis included demographic features: age and sex; features of index myocarditis hospitalization: duration, intensive care unit (ICU) level care, private facility, and rurality; category of myocarditis: reactive myocarditis, idiopathic myocarditis, autoimmune myocarditis, and malignant myocarditis; and Charlson comorbidity index and comorbidities which were listed above for the conditional logistic regression analysis. These covariates therefore included time invariant conditions as well as acute clinical events punctuated by hospitalizations coded as time-dependent covariates that remained positive for a 30-day period after they occurred. The reference group without these potential risk factors included both patients without the risk factor as well as times during which patients who had the risk factor were outside of the 30-day risk period. In addition to univariate analysis, multivariate analysis was performed whereby results were adjusted to three prespecified covariates: age, sex and Charlson comorbidity index. However, comorbidities included in the Charlson comorbidity index were not adjusted for the Charlson comorbidity index to avoid multicollinearity.

The same multivariate analysis was performed using the Fine and Gray method where the primary outcome of interest was recurrence and death was the competing event. The associations between myocarditis recurrence and the various possible risk factors were quantified with subdistribution hazards. As the Fine and Gray method does not have a standard approach to incorporate time-dependent covariates, the analysis was only performed for time independent covariates.

An exploratory multivariate Cox regression analysis was also performed without prespecified covariates. In this exploratory analysis, variables were included according to forward selection if their *P*-value in the multivariable model was significant (*P* < 0.05) and removed with a backward selection method if their *P*-value in the multivariable model was less than 0.1. Variables with small sample sizes that failed to converge during model creation were excluded.

## Results

Most of the study cohort was male (66.1%), with a median age of 42 years (*Table 1*). Myocarditis patients were moderately comorbid with a median Charlson comorbidity index of 1, with 15% requiring ICU level care. Of the 4071 patients admitted with myocarditis, 182 (4.5%) patients died in-hospital while a further 632 (15.5%) patients died post-discharge during the study period, with 124 (3.0%) patients having had their first recurrent myocarditis at a median follow-up of 73 [interquartile range (IQR) 20–403] days. Patients with recurrent myocarditis were younger than those without recurrence, stayed 1.4 days longer in hospital, and had fewer ICU admissions and fewer comorbidities during their index myocarditis admission. Median follow-up after index myocarditis diagnosis was 4.8 years (IQR 1.6–8.9 years, maximum 17.2 years).

**Table 1:** Summary features of study cohort during index myocarditis admission

Feature	Entire cohort (*n* = 4071)	Recurrent myocarditis^a^ (*n* = 124)	No recurrent myocarditis (*n* = 3947)	*P*-value
Demographics				
Male	66.1% (2690/4071)	66.9% (83/124)	66.1% (2607/3947)	0.913
Age (years)	42.4 (27.2–58.7)	37.6 (21.8–49.3)	42.6 (27.5–58.8)	<0.001
Private hospital	8.2% (333/4071)	2.4% (3/124)	8.4% (330/3947)	0.027
Major city^b^	74.2% (3007/4055)	73.4% (91/124)	74.2% (2916/3931)	0.925
Rural^c^	5.8% (235/4055)	4.8% (6/124)	5.8% (229/3931)	0.789
Background conditions				
Heart failure	23.5% (955/4071)	26.6% (33/124)	23.4% (922/3947)	0.463
Acute myocardial infarction	15.7% (340/4071)	16.9% (21/124)	15.7% (619/3947)	0.801
Atrial fibrillation	12.5% (507/4071)	10.5% (13/124)	12.5% (494/3947)	0.592
Diabetes	10.4% (423/4071)	4% (5/124)	10.6% (418/3947)	0.027
Malignancy	7.9% (321/4071)	8.1% (10/124)	7.9% (311/3947)	1.000
Ventricular arrhythmia	6.2% (253/4071)	9.7% (12/124)	6.1% (241/3947)	0.152
Autoimmunity	5.6% (229/4071)	7.3% (9/124)	5.6% (220/3947)	0.546
Pericarditis	5.6% (227/4071)	5.6% (7/124)	5.6% (220/3947)	1.000
Chronic kidney disease	4.6% (187/4071)	4% (5/124)	4.6% (182/3947)	0.932
Pulmonary hypertension	2.1% (86/4071)	0.8% (1/124)	2.2% (85/3947)	0.478
Charlson comorbidity index	1 (0–3)	1 (0–2)	1 (0–3)	0.026
Admission outcomes				
Admission duration (days)	4.9 (2.4–13.1)	4.7 (2.9–11.2)	4.9 (2.4–13.2)	0.953
ICU admission	15.4% (625/4071)	10.5% (13/124)	15.5% (612/3947)	0.161

Continuous variables reported as median [interquartile (IQR)]. Binary variables reported as percentage (frequency). Where denominators were less than 4071, this indicated missing data. Comparisons were made by the Mann–Whitney *U* test for continuous variables and the χ² test for categorical variables.

^a^Recurrent myocarditis was defined as an admission after the index inpatient spell where myocarditis was the primary diagnosis. The characteristics shown in *Table 1* were based on patient’s index myocarditis admission.

^b^Major city defined by Australian Statistical Geography Standard of 1.

^c^Rural defined by Australian Statistical Geography Standard of 3 or more

**Figure 1:**
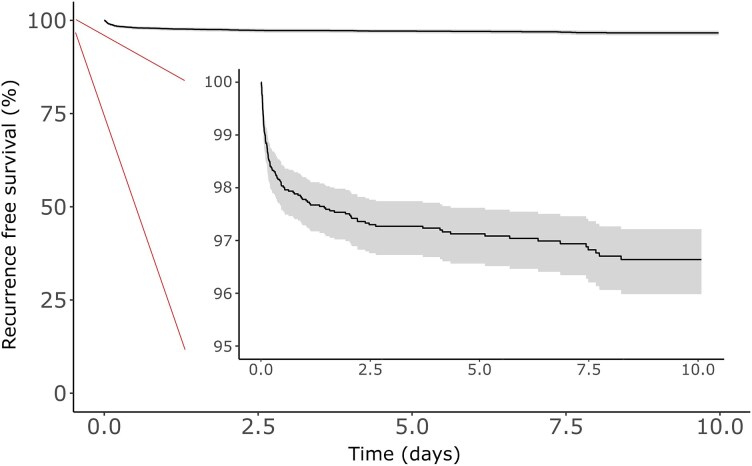
Survival free of recurrence of myocarditis after index admission for myocarditis with adjustment for competing risk of death. Analysis according to Fine and Gray shaded area indicates 95% confidence intervals.

More than half of myocarditis recurrence in the study cohort occurred within 3 months of the index myocarditis admission. The 30-day recurrence rate was 1.0%, the 6-month recurrence rate was 2.0%, the 1-year recurrence rate was 2.2%, the 5-year recurrence rate was 2.9%, and the 10-year recurrence rate was 3.4% (*Figure 1*).

**Figure 2:**
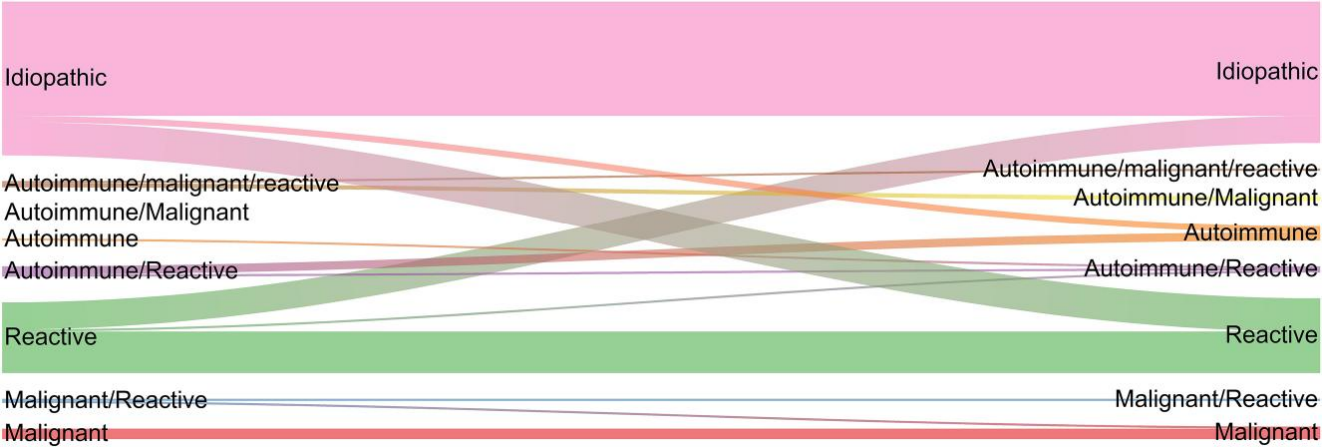
River plot of classification of myocarditis at index presentation compared to first recurrence for patients who had recurrent myocarditis. Idiopathic myocarditis was defined as the absence of autoimmunity, malignancy and reactive myocarditis.

Patients tended to keep the same classification of myocarditis (autoimmune, malignant, or reactive) during their recurrent admission compared to their index admission. Although a small proportion of patients with idiopathic myocarditis crossed over into different groups, including reactive myocarditis (*n* = 16) and autoimmune myocarditis (*n* = 3), the majority remained idiopathic (*n* = 55) (*Figure 2*).

### Risk factors for index myocarditis

Conditional logistic regression demonstrated several conditions were temporally associated with myocarditis within the following month (*Table 2*).

**Table 2:** Risk of myocarditis within 1 month of prespecified conditions

Primary diagnosis for hospitalizations in the 1-month preceding index myocarditis admission	Number of cases prior to myocarditis^a^	Odds ratio (95% CI)	*P*-value
Myocardial infarction	308	45.0 (28.2–71.7)	<0.0001
Pericarditis	74	26.2 (10.8–63.4)	<0.0001
Heart failure	200	18.3 (2.1–158.5)	<0.0001
Influenza	24	13.9 (8.3–23.4)	<0.0001
Ventricular arrhythmia	39	12.4 (3.9–39.5)	<0.0001
COVID-19	59	10.4 (4.7–22.7)	<0.0001
Respiratory disease	635	8.2 (5.6–11.9)	<0.0001
Autoimmune disease	134	6.1 (3.1–12.1)	<0.0001
Atrial fibrillation	136	6.0 (2.8–12.9)	<0.0001
Myositis	23	5.5 (1.2–25.1)	0.0009
Sarcoidosis	9	5.2 (1.4–18.5)	0.0002
Stroke	47	4.7 (0.6–36.2)	0.0257
Systemic lupus erythematosus	16	3.8 (0.4–33.3)	0.0734
Digestive disease	1062	2.9 (1.8–4.7)	<0.0001
Diabetes	74	2.0 (0.2–18.8)	0.3641
Malignancy	265	1 (0.4–2.7)	0.8987

Odds of risk factors up to 30 days prior to myocarditis admission compared to 31–390 days prior to myocarditis admission using conditional logistic regression.

^a^Number of patients with hospitalizations for each condition at any time prior to myocarditis, not just 30 days prior. Conditions required to be primary diagnosis for the hospital admission, except for COVID-19 which was based on secondary diagnosis.

The frequency histograms of these conditions show the same findings graphically (see Supplementary material online, *Figure S2*).

Sensitivity analyses were performed with different cases and control groups. The presence of risk factors in the month prior to myocarditis diagnosis was compared to a control group over the preceding year (31–390 days prior to myocarditis) in *Table 2*. If the control group was more distant, that is, from 360 to 720 days prior to myocarditis diagnosis, similar trends were observed but with larger effect sizes (see Supplementary material online, *Table S3*). The presence of risk factors within 3 months of myocarditis admission was also compared to a control group over the preceding year which showed similar albeit weaker trends (see Supplementary material online, *Table S4*). By contrast, as a negative control, the presence of potential risk factors of myocarditis 90–120 days prior to myocarditis admission was compared to 121–480 days prior to myocarditis admission, and as expected, the associations of the prespecified risk factors for myocarditis were weaker when they were more temporally distant (see Supplementary material online, *Table S5*).

Analysis was repeated with different conditions to further verify this methodology (see Supplementary material online, *Tables S6* and *S7*). The positive control cohort pericarditis associated with similar conditions as myocarditis (see Supplementary material online, *Table S8*). The associations with hip fracture repair were used as a negative control cohort and potential myocarditis risk factors had almost no association with hip fracture repair using the same analytical techniques (see Supplementary material online, *Table S9*).

The risk of myocarditis related to common presentations for infectious diseases was also assessed. The risk of myocarditis was highest after episodes of meningoencephalitis. Endocarditis, pneumonia, and common viral respiratory pathogens were also strongly associated with myocarditis. However, osteomyelitis, gastroenteritis, and urinary tract infections were less strongly associated with myocarditis (*Table 3*).

**Table 3:** Risk of myocarditis within 1 month of admissions for common infections

Infection	Number of cases prior to myocarditis^a^	Odds ratio (95% CI)	*P*-value
Meningoencephalitis	19	39.2 (3.7–415.4)	<0.0001
Endocarditis	13	18.3 (2.1–158.5)	<0.0001
Influenza	24	16 (3.3–78.1)	<0.0001
Bacterial pneumonia	201	14.2 (7.4–27.6)	<0.0001
COVID-19	59	10.4 (4.7–22.7)	<0.0001
Cellulitis	97	6.7 (1.7–27.2)	<0.0001
Upper respiratory tract infection^b^	46	6.6 (1.4–30.7)	0.0003
Osteomyelitis	10	6 (0.2–218.5)	0.1435
Gastroenteritis	127	3.1 (1–9.4)	0.003
Urinary tract infection	85	2.4 (0.4–15.8)	0.1645

Risk of myocarditis 30 days after admission compared to 31–390 days after admission using conditional logistic regression.

^a^Cases of infection at any time prior to myocarditis, not just 30 days prior. Conditions required to be primary diagnosis for the hospital admission, except for COVID-19 which was based on secondary diagnosis.

^b^Upper respiratory tract infection for undiagnosed organism, therefore excluding COVID-19 and influenza. Risk of myocarditis 30 days after admission compared to 31–390 days after admission using conditional logistic regression.

### Risk factors for recurrent myocarditis

Patients with a younger age during the index myocarditis diagnosis admission were more likely to experience myocarditis recurrence, although sex did not influence recurrence risk (*Table 4*). An admission to the intensive care unit was associated with a numerically but not statistically significant reduction in the recurrence of myocarditis. The types of myocarditis (reactive, autoimmune, malignant, or idiopathic) did not associate with the risk of myocarditis recurrence. Furthermore, no comorbidities were found to significantly change the risk of myocarditis recurrence, including when they were aggregated into the Charlson comorbidity index. Similar results were found with cause specific Cox regression analysis and irrespective of whether adjustment was performed (see Supplementary material online, *Table S10*).

**Table 4:** Predictors of myocarditis recurrence according to competing risk analysis

Variables	Total count (e.g. number of males in cohort)	Total count with recurrent myocarditis (e.g. number of males in cohort who have recurrent myocarditis)	Subdistribution hazard ratio (95% CI)	*P*-value
Demographics at time of index myocarditis admission				
Sex (male)	2690	83	0.93 (0.64–1.36)	0.70
Age (per 10 years)	4071	124	0.84 (0.75–0.94)	0.002
Hospitalization characteristics at time of index myocarditis admission				
Admission duration (weeks)	4071	124	1.00 (0.97–1.02)	0.710
Admitted to private hospital	333	3	0.32 (0.10–1.02)	0.054
Intensive care unit admission	625	13	0.54 (0.29–1.00)	0.052
Hospital in major city^a^	3007	91	0.95 (0.64–1.42)	0.81
Hospital in rural area^b^	235	6	0.85 (0.37–1.92)	0.69
Comorbidities at time of index myocarditis admission				
Charlson comorbidity index	4071	124	1.03 (0.92–1.15)	0.62
Idiopathic myocarditis^c^	2317	74	0.97 (0.66–1.42)	0.88
Digestive disease	1732	55	1.37 (0.93–2.02)	0.11
Respiratory disease	1695	44	0.83 (0.55–1.24)	0.36
Reactive myocarditis^d^	1537	44	1.00 (0.68–1.47)	0.99
Heart failure	955	33	1.40 (0.93–2.12)	0.10
Myocardial infarction	640	21	1.48 (0.91–2.39)	0.11
Atrial fibrillation	507	13	1.16 (0.59–2.28)	0.66
Diabetes	423	5	0.47 (0.19–1.15)	0.098
Malignancy	321	10	1.64 (0.81–3.31)	0.17
COVID-19	301	5	0.71 (0.29–1.73)	0.45
Ventricular arrhythmia	253	12	1.87 (1.00–3.49)	0.051
Autoimmune disease	229	9	1.52 (0.76–3.03)	0.23
Pericarditis	227	7	0.92 (0.43–1.98)	0.84
Influenza	136	3	0.80 (0.25–2.55)	0.71
Stroke	89	2	1.01 (0.25–4.14)	0.99
Myositis	62	2	1.16 (0.28–4.73)	0.84
Systemic lupus erythematosus	50	1	0.61 (0.08–4.35)	0.62
Sarcoidosis	32	2	2.65 (0.66–10.59)	0.17

All analysis were adjusted for age, sex, and Charlson comorbidity index and competed against death according to the Fine and Gray method; however, comorbidities included in the Charlson comorbidity index were not adjusted for this.

^a^Australian Statistical Geography Standard 1.

^b^Australian Statistical Geography Standard ≥ 3.

^c^Idiopathic myocarditis defined as the absence of reactive myocarditis or the comorbidities autoimmune disease or malignancy.

^d^Reactive myocarditis defined as having respiratory or digestive system illness at presentation or requiring hospitalization in the past 30 days.

During follow-up, hospitalizations for certain conditions were associated with an increased risk of myocarditis for the next 30 days including COVID-19 infection, ventricular arrhythmia, pericarditis, diabetes, and autoimmune diseases including sarcoidosis. Although respiratory diseases in general were associated with a recurrence of myocarditis over the next 30 days, this was entirely attributable to COVID-19: all four cases of recurrent myocarditis after a respiratory disease were when this respiratory disease was COVID-19 (*Table 5*). Despite significant results being found, these findings should be interpreted with caution and considered exploratory due to the rarity of myocarditis recurrence. Confidence intervals for the hazard of myocarditis after re-hospitalization were large due to the small numbers.

**Table 5:** Risk of myocarditis recurrence within 30 days of hospitalization for other conditions

Cause of hospitalization	Number of hospitalizations after index myocarditis admission	Number of myocarditis recurrences within 30 days of hospitalization	Hazard ratio (95% CI)	*P*-value
Respiratory disease	808	4	3.75 (1.3–10.77)	0.014
Digestive disease	784	1	1.99 (0.28–14.35)	0.495
COVID-19	406	4	12.42 (4.4–35.08)	<0.001
Heart failure	398	2	1.81 (0.44–7.45)	0.409
Malignancy	255	0	–	–
Myocardial infarction	234	2	1.9 (0.45–8.03)	0.383
Atrial fibrillation	171	1	3.36 (0.45–25.32)	0.239
Autoimmune disease	170	2	6.65 (1.55–28.6)	0.011
Ventricular arrhythmia	82	2	9.63 (2.25–41.32)	0.002
Stroke	82	0	–	–
Pericarditis	79	4	13.95 (4.52–43.09)	<0.001
Diabetes	63	1	33.66 (4.18–271.38)	<0.001
Influenza	38	0	–	–
Myositis	27	1	6.19 (0.86–44.78)	0.071
Systemic lupus erythematosus	17	0	–	–
Sarcoidosis	8	1	41.38 (4.3–398.56)	0.001

All analyses were multivariable Cox regression analysis with adjustment for age, sex, and Charlson comorbidity index; however, comorbidities included in the Charlson comorbidity index were not adjusted for the Charlson comorbidity index. Hospitalizations during follow-up were treated as time-dependent covariates and were categorized according to the primary diagnosis during that admission. If the model did not converge, the hazard ratio for the respective diagnosis was not displayed, and in all cases, this was because there were no hospitalizations within 30 days of the hospitalization for the respective diagnosis. There were 124 cases of myocarditis recurrence in the entire cohort.

**Figure 3:**
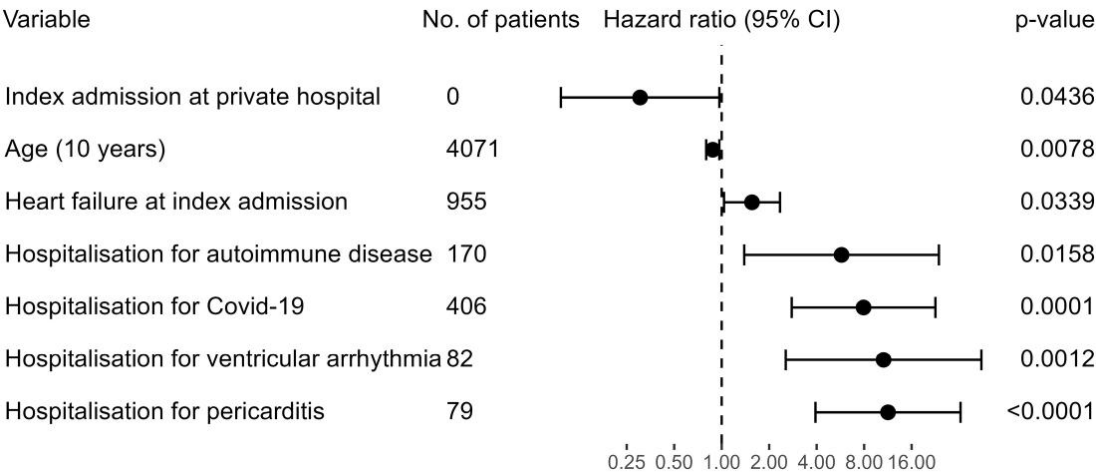
Prediction of myocarditis recurrence in Cox multivariable regression model. Model derived by forward and backward selection from prespecified variables. Myocarditis risk was assessed for 30 days after hospitalization.

In a further exploratory analysis, where the above variables (in *Tables 4* and *5*) were put through a forward and backward selection algorithm, few variables were found to predict myocarditis recurrence (*Figure 3*). Although in the primary analysis (*Table 4*), no comorbidities were predictive of myocarditis recurrence, in this exploratory multivariable analysis, heart failure diagnosed at or prior to index hospitalization was predictive of recurrence. New re-hospitalizations had larger effects on the risk of recurrence with autoimmune disease, COVID-19, ventricular arrhythmia, and pericarditis admissions all being harbingers of recurrent myocarditis, increasing the risk of recurrent myocarditis between 5 and 10-fold (*Figure 3*). Older age reduced the risk of myocarditis recurrence. This multivariable regression model met collinearity assumptions (see Supplementary material online, *Table S11*). The concordance statistic for this model was 0.60.

## Discussion

### Main findings

The present study has quantified the contribution of classical risk factors to the incidence of myocarditis. This research indicates that although myocarditis is rare, it is much more common after a recent hospital presentation for heart failure, arrhythmia, respiratory diseases including COVID-19, and various autoimmune diseases. The effect size of these presentations was large with odds ratios between 5 and 50. The same risk factors predict myocarditis recurrence in patients with a known background history of myocarditis. However, in these patients with a known history of myocarditis, features of their index admission were poorly predictive of myocarditis recurrence.

### Previous research

There has been previous research that also provides evidence of the risk factors of myocarditis listed in the present study. In recent years, a large amount of data including case series and cohort studies have demonstrated the strong epidemiological association between COVID-19 and myocarditis.^18^ Previous cohort studies have demonstrated similarly to the present study that younger age was predictive of myocarditis recurrence.^11,19^ Ventricular arrhythmia has also previously been found to be predictive of myocarditis recurrence.^11^ By contrast, in the present study, although ventricular arrhythmia was predictive of either index or recurrent myocarditis occurring in the next 30 days, ventricular arrhythmia at index presentation tended towards more recurrence but this was not a significant finding (*P* = 0.051). Other features have utility for predicting myocarditis including chronic obstructive pulmonary disease, inflammatory bowel disease, late gadolinium enhancement, and left ventricular ejection fraction.^11,19^ Unfortunately, the present study could not corroborate the importance of imaging as these data were not available.

Although various infectious diseases have been temporally related to myocarditis, no previous studies have compared the risk imputed from different infectious diseases. The association between respiratory viral infections, including COVID-19 and myocarditis, is well known and is reinforced in the present study.^6,7^ The present study also justifies the lack of reported associations between myocarditis and other common infectious diseases such as urinary tract infection and osteomyelitis. However, through the first systemic analysis of risk factors in a state-wide cohort, it also raises the hypothesis that meningoencephalitis and endocarditis, though relatively less common, also appear to be strongly related to myocarditis. These connections have not previously been well established although they have been shown in case reports.^20,21^

Prior autopsy studies reported the largest histological type of myocarditis is lymphocytic secondary to an infection.^4,5^ Moreover, a cross-sectional study of death certification demonstrated a relatively high rate of infection (15%), cardiac disease (12.5%), and a smaller burden of autoimmune disease (2%).^22^ However, to the best of our knowledge, there are no longitudinal studies of large populations to compare with the findings of the present study which identify risk factors that are temporally related to myocarditis diagnosis.

Genetic information is also critical for the prognostication of myocarditis as the genetic milieu is known to influence the development of dilated cardiomyopathy.^23^ For example, children with acute myocarditis, many with proven viral aetiology, have been found to have very high rates of cardiomyopathy genes.^24^ However, laboratory and genetic data were not available in the present study to expand on this previous seminal work.

### Current study

Index cases of myocarditis were found to be strongly temporally related to myocardial infarction, pericarditis, heart failure, atrial fibrillation, ventricular arrhythmia, respiratory diseases including COVID-19, and various autoimmune diseases. The present study does provide some hypothesis generating exploratory analysis that the risk of myocarditis differs based on the type of infection. In this study, respiratory viral infections, meningoencephalitis, and endocarditis imputed higher risk; but gastroenteritis, osteomyelitis, and urinary tract infections were either weakly or not associated with myocarditis. Given the large confidence intervals and the lack of corroborating alternative studies, these results however should be considered speculative.

In the conditional logistic regression analysis which identified risk factors for the index case of myocarditis, patients acted as their own controls. This obviated the need for further adjustment for confounders such as demographics or comorbidities. These findings were verified with sensitivity analysis. The methods were also tested with positive and negative controls which as expected demonstrated associations of similar risk factors for pericarditis as with myocarditis, but not for hip fracture repair.

The association between myocarditis and some cardiac presentations was unexpected. It was surprising that myocarditis occurrence was related to myocardial infarction given that coronary artery disease can be considered an exclusionary criterion for myocarditis diagnosis.^25^ It is possible that the overlapping troponinaemia and clinical features of both conditions can lead to diagnostic confusion. For example, it has been found across multiple hospitals in the UK that 13% of COVID-19 patients with myocardial injury had an infarct appearance on cardiac magnetic resonance imaging.^26^ In 40% of cases of myocarditis diagnosis in the present study, a diagnostic coronary angiogram was performed during the index admission or prior to admission; however, other forms of assessment of the coronary arteries to exclude ischaemic heart disease were not available in the APDC.

The risk of myocarditis recurrence was also temporally related to the same risk factors as for the index diagnosis of myocarditis with a notable exception of myocardial infarction which was not a risk factor for myocarditis recurrence. The risk factors for myocarditis recurrence were tested by Cox regression analysis which allowed adjustment for various confounders including demographics and comorbidities. The findings were also replicated with a completely different methodology Fine and Gray analysis which additionally adjusted for competing risks, given many patients died instead of having recurrent myocarditis. Myocarditis recurrence was rare with only 178 cases during a median 4.8 years of follow-up and so the numbers of patients with myocarditis occurrence temporally related to any specific diagnosis were small, generating large confidence intervals. Features of index myocarditis admission were only weakly related to myocarditis recurrence, but recurrence was slightly more common in younger patients admitted for a longer duration but was slightly less common in patients requiring ICU during their index admission. Interestingly, there was no signal that the aetiology of myocarditis, whether post-infective, autoimmune, or associated with a malignancy, was relevant in predicting recurrence. Admittedly, this did not factor in any follow-up or immunosuppression instituted for these patients.

In multivariable analysis, there were no features of the index admission for myocarditis other than age and the presence of heart failure that predicted subsequent recurrence. Notably, the presence of neither autoimmune disease nor malignancy was associated with recurrence. There are imaging findings including reduction in systolic function and late gadolinium enhancement that have been shown in other studies to be predictive of recurrence.^27^ However, the findings of the present study caution against using comorbidities to risk stratify patients for ongoing surveillance.

Myocarditis is a difficult diagnosis and the time that myocarditis was diagnosed by clinicians may be disparate from the time of onset of clinical manifestations of myocarditis.^2^ Given the rarity of myocarditis, it is often missed in the differential diagnosis list and the clinical and laboratory findings of myocarditis are largely non-specific. Although endomyocardial biopsy remains the diagnostic gold standard, the vast majority of patients are diagnosed without the histopathological evidence of myocarditis required for a definitive diagnosis.^25^ In a prospective study, the median duration of symptoms prior to in hospital diagnosis was 0.5 months (IQR 0–3 months).^28^ It is therefore likely that many admissions for heart failure, pericarditis, or arrhythmia prior to the diagnosis of myocarditis were early disease manifestations rather than risk factors as they are typically understood. The observational study design cannot delineate the mechanism of the associations, and it is possible that many patients presenting with cardiac complaints had yet undiagnosed myocarditis. Regardless of the underlying cause, this study’s finding that presentations such as respiratory illnesses, arrhythmias, heart failure, pericarditis, and autoimmune disease are temporally associated with myocarditis suggests that the presence of these features should raise clinical suspicion for myocarditis.

### Limitations

This study has several important limitations. Firstly, myocarditis risk was assessed within a 30-day interval following the emergence of potential risk factors. The choice of this timeframe was informed by prior evidence: (i) myocarditis risk is higher 28 days after COVID-19 vaccination,^29^ (ii) basic science data indicate a time course of 1–7 days of viral replication followed by 1–4 weeks of immune mediated injury,^30,31^ (iii) the typical prodromal period of a few weeks,^32^ and (iv) previous observational studies showing that a 3-day window captures a large proportion of myocarditis risk factors.^33^ Consistent with these findings, we observed an increased incidence of potential risk factors in the 20–30 days preceding myocarditis diagnosis (see Supplementary material online, *Figure S2*). Nevertheless, this approach does not account for chronic or long-standing exposures. For example, malignancy may confer risk over extended periods and hospitalization for malignancy does not necessarily coincide with the timing of chemotherapy administration. This is reflected in the lack of a significant association between malignancy and myocarditis in our regression analysis.

Secondly, this study relied on administrative data and disease classification was based on diagnostic coding. The accuracy of myocarditis diagnoses therefore depends on the quality of clinical documentation and coding practices. While a Swedish validation study reported a positive predictive value of 96% for myocarditis in a similar administrative dataset, misclassification remains possible.^34^ Only hospitalized cases were included, meaning that recurrent myocarditis diagnosed in outpatient settings was not captured. Furthermore, the dataset lacked laboratory test results, medication history, and imaging findings including echocardiography and cardiac magnetic resonance. This precluded detailed phenotyping of myocarditis with respect to triggers, inflammatory burden, genetics, or ventricular function. Endomyocardial biopsy was performed in only 6% of patients and biopsy results were unavailable. As a result, differentiation between distinct myocarditis subtypes such as viral myocarditis, autoimmune myocarditis, giant cell myocarditis, or inflammatory phases of arrhythmogenic cardiomyopathy was not possible. Given this heterogeneity, the findings of the present study cannot be readily extrapolated to specific clinical subgroups.

Finally, although we undertook multiple strategies to test the robustness of our findings including validation against alternative time windows, sensitivity analyses using positive and negative controls, and reproducing findings with different statistical techniques, these methods have limitations. Although the controls exhibited expected behaviour, they differed in relevant ways from the myocarditis cohort. Pericarditis, for example, has different risk factors from myocarditis and hip fracture patients differ substantially from myocarditis patients with respect to age and comorbidity burden. External validation in independent international cohorts will therefore be essential to confirm the generalisability of our results.

## Conclusions

In summary, we reported the risk factors of first episode of myocarditis and myocarditis recurrence. Respiratory diseases, especially COVID-19, arrhythmias, heart failure, and autoimmune disease were all associated with myocarditis which identifies them as possible risk factors or symptoms of myocarditis presentations. Nevertheless, myocarditis is not a common disease and even patients with a known history of myocarditis rarely experience recurrence.

## Supplementary Material

oeaf130_Supplementary_Data

## Data Availability

The original data presented in the study are available in NSW Admitted Patient Data Collection (APDC) database and the NSW Registry of Births, Deaths & Marriages. These data can be accessed by application to the NSW government.
