# Supplementary material for: Risk factors for myocarditis hospitalization and recurrence: a state-wide retrospective observational study
Source: Eur Heart J Open. 2025 Oct 9;5(6):oeaf130. doi: 10.1093/ehjopen/oeaf130 (PMC12604094; doi:10.1093/ehjopen/oeaf130)
Supplement: oeaf130_Supplementary_Data [file oeaf130_supplementary_data.docx]

**Supplementary files**

**Supplementary Table 1: Inclusion and exclusion criteria, starting with 5968 patients with a diagnosis of myocarditis in the Admitted Patient Data Collection**

| Exclusionary criteria | Number excluded | Number remaining |
| --- | --- | --- |
| No exclusions from all myocarditis admissions to NSW hospitals 2001 to 2022 | 0 | 5968 |
| Not resident of NSW | 183 | 5783 |
| Lacking 6 months follow up (i.e. admitted after September 30, 2021) | 387 | 5396 |
| Lacking 3 months of background data (i.e. admitted before July 1, 2004) | 484 | 4912 |
| Recurrent admission for myocarditis (not the index case) | 841 | 4071 |

**Supplementary Table 2: The International Classification of Disease (ICD-10AM) codes used in the present study**

| Diagnosis | ICD-10AM or ACHI code |
| --- | --- |
| Atrial fibrillation | I48, I48.0, I48.1, I48.2, I48.3, I48.4, I48.9 |
| AIDS | B20, B21, B22, B23, B23.0, B23.8, B24 |
| Chronic kidney disease | N18, N18.3, N18.4, N18.5, N18.9, N19, I12.0, I13.1, U87.1, Z49, Z49.0, Z49.1, Z49.2, Z94.0, Z99.2 |
| Covid | U06.0, U07.1, U07.2, U07.7 |
| CTD | M30, M30.0, M30.1, M30.2, M30.3, M30.8, M31, M31.0, M31.1, M31.2, M31.3, M31.4, M31.5, M31.6, M31.7, M31.8, M31.9, M32, M32.0, M32.1, M32.8, M32.9, M33, M33.0, M33.1, M33.2. M33.9, M34, M34.0, M34.1, M34.2, M34.8, M34.9, M35, M35.0, M35.1, M35.2, M35.3, M35.4, M35.5, M35.6, M35.7, M35.8, M35.9, M36, M36.0, M36.1, M36.2, M36.3, M36.4, M36.8 |
| Dilated cardiomyopathy | I42.0 |
| Dementia | F00, F00.0, F00.1, F00.2, F00.9, F01, F01.0, F01.2, F01.3, F01.8, F01.9, F02, F02.0, F02.1, F02.2, F02.3, FO2.4, FO2.8, F03, F05.1, U79.1, G30, G30.0, G30.1, G30.8, B30.9 |
| Diabetes | E10, E10.0, E10.00, E10.01, E10.02, E10.1, E10.10, E10.11, E10.12, E10.13, E10.14, E10.15, E10.16, E10.2, E10.20, E10.21, E10.22, E10.23, E10.29, E10.3, E10.30, E10.31, E10.32, E10.33, E10.34, E10.35, E10.36, E10.39, E10.4, E10.40, E10.41, E10.42, E10.43, E10.49, E10.5, E10.50, E10.51, E10.52, E10.53, E10.59, E10.6, E10.60, E10.61, E10.62, E10.63, E10.64, E10.65, E10.69, E10.7, E10.70, E10.71, E10.73, E10.8, E10.80, E10.81, E10.9, E10.90, E10.91, E11, E11.0, E11.00, E11.01, E11.02, E11.1, E11.10, E11.11, E11.12, E11.13, E11.14, E11.15, E11.16, E11.2, E11.20, E11.21, E11.22, E11.23, E11.29, E11.3, E11.30, E11.31, E11.32, E11.33, E11.34, E11.35, E11.36, E11.39, E11.4, E11.40, E11.41, E11.42, E11.43, E11.49, E11.5, E11.50, E11.51, E11.52, E11.53, E11.59, E11.6, E11.60, E11.61, E11.62, E11.63, E11.64, E11.65, E11.69, E11.7, E11.70, E11.71, E11.72, E11.73, E11.8, E11.80, E11.81, E11.9, E11.90, E11.91, E12, E12.0, E12.00, E12.01, E12.1, E12.10, E12.11, E12.2, E12.20, E12.21, E12.3, E12.30, E12.31, E12.4, E12.40, E12.41, E12.5, E12.50, E12.51, E12.6, E12.60, E12.61, E12.7, E12.70, E12.71, E12.8, E12.80, E12.81, E12.9, E12.90, E12.91, E13, E13.0, E13.00, E13.01, E13.02, E13.1, E13.10, E13.11, E13.12, E13.13, E13.14, E13.15, E13.16, E13.2, E13.20, E13.21, E13.22, E13.23, E13.29, E13.3, E13.30, E13.31, E13.32, E13.33, E13.34, E13.35, E13.36, E13.39, E13.4, E13.40, E13.41, E13.42, E13.43, E13.49, E13.5, E13.50, E13.51, E13.52, E13.53, E13.59, E13.6, E13.60, E13.61, E13.62, E13.63, E13.64, E13.65, E13.69, E13.7, E13.70, E13.71, E13.72, E13.73, E13.8, E13.80, E13.81, E13.9, E13.90, E13.91, E14, E14.0, E14.00, E14.01, E14.02, E14.1, E14.10, E14.11, E14.12, E14.13, E14.14, E14.15, E14.16, E14.2, E14.20, E14.21, E14.22, E14.23, E14.29, E14.3, E14.30, E14.31, E14.32, E14.33, E14.34, E14.35, E14.36, E14.39, E14.4, E14.40, E14.41, E14.42, E14.43, E14.49, E14.5, E14.50, E14.51, E14.52, E14.53, E14.59, E14.6, E14.60, E14.61, E14.62, E14.63, E14.64, E14.65, E14.69, E14.7, E14.70, E14.71, E14.72, E14.73, E14.8, E14.80, E14.81, E14.9, E14.90, E14.91 |
| Emphysema | J41, J41.0, J41.1, J41.8, J42, J43, J43.0, J43.1, J43.2, J43.8, J43.9, J44, J44.0, J44.1, J44.8, J44.9, J45, J45.0, J45.1, J45.8, J45.9, J46, J47, U83.1, U83.2, U83.3, J98.2, J98.3 |
| Heart failure | I11, I11.0, I13.0, I13.1, I13.2, I42, I43, I42, I42.0, I42.1, I42.2, I42.3, I42.4, I42.5, I42.6, I42.7, I42.8, I42.9, I43, I43.0, I43.1, I43.2, I43.8, I50, I50.0, I50.9, I50.1, I13.53, I14.53, I25.5 #NB: does not include U82.2, E10.53, E11.53 |
| Hemiplegia | G80.02, G80.1, G80.2, G81, G81.0, G81.1, G81.9, G82, G82.0, G82.00, G82.01,G82.02, G82.03, G82.04, G82.05, G82.06, G82.1, G82.10, G82.11, G82.12,G82.13, G82.14, G82.15, G82.16, G82.2, G82.20, G82.21, G82.22, G82.23,G82.24, G82.25, G82.26, G82.3, G82.30, G82.31, G82.32, G82.33, G82.34,G82.35, G82.36, G82.4, G82.40, G82.41, G82.42, G82.43, G82.44, G82.45,G82.46, G82.5, G82.51, G82.52, G82.53, G82.54, G82.55, G82.56,U80.5, G11.4, M62.3, M62.30, M62.31, M62.32, M62.33, M62.34, M62.35, M62.36, M62.37, M62.38, M62.39 |
| HIV | B20, B21, B22, B23, B23.0, B23.8, B24, F02.4, O98.7, R75, Z21 |
| Hypertension | I10, I11, I11.0, I11.9, I12, I12.0, I12.9, I13, I13.0, I13.1, I13.2, I13.9, I15, I15.0, I15.1, I15.2, I15.8, I15.9, U82.3 |
| Hyperlipidaemia | E78, E78.0, E78.1, E78.2, E78.3, E78.4, E78.5, E78.6, E78.8, E78.9 |
| Inflammatory bowel disease | K50, K50.0, K50.1, K50.8, K50.9, K51, K51.0, K51.2, K51.3, K51.4,K51.5, K51.8, K51.9, M07.4, M07.40, M07.41, M07.42, M07.43, M07.44,M07.45, M07.46, M07.47, M07.48, M07.49, M07.5, M07.50, M07.51,M07.52, M07.53, M07.54, M07.55, M07.56, M07.57, M07.58, M07.59,M07.6, M07.61, M07.62, M07.63, M07,64, M07.65, M07.66, M07.67,M07.68, M07.69, U84.1, U84.2, M09.1, M09.10, M09.11, M09.12,M09.13, M09.14, M09.15, M09.16, M09.17, M09.18, M09.19, M09.2,M09.20, M09.21, M09.22, M09.23, M09.24, M09.25, M09.26, M09.27,M09.28, M09.29 |
| Ischaemic heart disease | I20, I20.0, I20.1, I20.8, I20.9, I21, I21.0, I21.1, I21.2, I21.3, I21.4, I21.9, I22, I22.0, I22.1, I22.8, I22.9, I23, I23.0, I23.1, I23.2, I23.3, I23.4, I23.5, I23.6, I23.8, I24, I24.0, I24.1, I24.8, I24.9, I25, I25.0, I25.1, I25.10, I25.11, I25.12, I25.13, I25.2, I25.3, I25.4, I25.5, I25.6, I25.8, I25.9 |
| Influenza | J09, J10, J10.0, J10.1, J10.8, J11, J11.0, J11.1, J11.8 |
| Leukaemia | C90.1, C90.10, C90.11, C91, C91.0, C91.00, C91.01, C91.1, C91.10, C91.11, C91.2, C91.20, C91.21, C91.3, C91.30, C91.31, C91.4, C91.40, C91.41, C91.5, C91.50, C91.51, C91.6, C91.60, C91.61, C91.7, C91.70, C91.71, C91.8, C91.80, C91.81, C91.9, C91.90, C91.91, C92, C92.0, C92.00, C92.01, C92.1, C92.10, C92.11, C92.2, C92.20, C92.21, C92.4, C92.40, C92.41, C92.5, C92.50, C92.51, C92.6, C92.60, C92.61, C92.7, C92.70, C92.71, C92.8, C92.80, C92.81, C92.9, C92.90, C92.91, C93, C93.0, C93.00, C93.01, C93.1, C93.10, C93.11, C93.2, C93.20, C93.21, C93.3, C93.30, C93.31, C93.7, C93.70, C93.71, C93.9, C93.90, C93.91, C94, C94.0, C94.00, C94.01, C94.2, C94.20, C94.21, C94.3, C94.30, C94.31, C94.7, C94.70, C94.71, C95, C95.0, C95.00, C95.01, C95.1, C95.10, C95.11, C95.2, C95.20, C95.21, C95.7, C95.70, C95.71, C95.9, C95.90, C95.91, D47.5, Z85.6, M9733/3, M9742/3, M980, M9800/3, M9801/3, M9802/3, M9803/3, M9804/3, M9805/3, M9806/3, M9807/3, M9808/3, M9809/3, M981-M983, M9811/3, M9812/3, M9813/3, M9814/3, M9815/3, M9816/3, M9817/3, M9818/3, M9820/3, M9821/3, M9822/3, M9823/3, M9824/3, M9825/3, M9826/3, M9827/3, M982-M983, M9830/3, M9831/1, M9832/3, M9833/3, M9834/3, M9835/3, M9836/3, M9837/3, M9840/3, M984-M993, M9850/3, M9860/3, M9861/3, M9862/3, M9863/3, M9864/3, M9865/3, M9866/3, M9867/3, M9868/3, M9869/3, M9870/3, M9871/3, M9872/3, M9873/3, M9874/3, M9875/3, M9876/3, M9880/3, M9890/3, M9891/3, M9892/3, M9893/3, M9894/3, M9895/3, M9896/3, M9897/3, M9898/3, M9900/3, M9910/3, M9911/3, M994, M9940/3, M9945/3, M9946/3, M9948/3, M9963/3, M9964/3 |
| Liver disease | K70, K70.0, K70.1, K70.2, K70.3, K70.4, K70.9, K71, K71.0, K71.1, K71.2, K71.3, K71.4, K71.5, K71.6, K71.7, K71.8, K71.9, K72, K72.0, K72.1, K72.9, K73, K73.0, K73.1, K73.2, K73.8, K73.9, K74, K74.0, K74.1, K74.2, K74.3, K74.4, K74.5, K74.6, K75,K75.0, K75.1, K75.2, K75.3, K75.4, K75.8, K75.9, K76, K76.0, K76.1, K76.2,K76.3, K76.4, K76.5, K76.6, K76.7, K76.8, K76.9,B18, B18.0, B18.1, B18.2, B18.8, B18.9, Z94.4,I85, I85.0, I85.9, I86.4, I98.2, I98.20, I98.21, I98.3 |
| Moderate to severe liver disease | K70.4, K71.1, K72, K72.0, K72.1, K72.9, K76.7, I85, I85.0, I85.9, I86.4, I98.2, I98.20, I98.21, I98.3 |
| Lymphoma | C81, C81.0, C81.1, C81.2, C81.3, C81.4, C81.7, C81.9, C82, C82.0, C82.1, C82.2, C82.3, C82.4, C82.5, C82.6, C82.7, C82.9, C83, C83.0, C83.1, C83.2, C83.3, C83.4, C83.5, C83.6, C83.7, C83.8, C83.9, C84, C84.2, C84.3, C84.4, C84.5, C84.6, C84.7, C84.8, C84.9, C85, C85.1, C85.2, C85.7, C85.9, C86, C86.0, C86.1, C86.2, C86.3, C86.4, C86.5, C88.4, C88.40, C88.41, C91.5, C91.50, C91.51, C96.3, M959, M9590/3, M9591/3, M9595/3, M9596/3, M9597/3, M959-M972, M9650/3, M9651/3, M9653/3, M9654/3, M9655/3, M9659/3, M965-M966, M9663/3, M9664/3, M9665/3, M9667/3, M9670/3, M9671/3, M9672/3, M9673/3, M9674/3, M9675/3, M9676/3, M9677/3, M9678/3, M9679/3, M967-M969, M967-M972, M9680/3, M9681/3, M9682/3, M9683/3, M9684/3, M9685/3, M9686/3, M9687/3, M9688/3, M9689/3, M969, M9690/3, M9691/3, M9692/3, M9693/3, M9694/3, M9695/3, M9696/3, M9697/3, M9698/3, M9699/3, M9702/3, M9703/3, M9704/3, M9705/3, M9706/3, M9707/3, M9708/3, M9709/3, M970-M971, M971, M9711/3, M9712/3, M9713/3, M9714/3, M9716/3, M9717/3, M9719/3, M972, M9723/3, M9725/3, M9726/3, M9727/3, M9728/3, M9729/3, M9735/3, M9737/3, M9738/3, C88.4, C88.40, C88.41 |
| Myocarditis | I01.2, I09.0, I40, I40.0, I40.1, I40.8, I40.9, I41, I41.0, I41.1, I41.2, I41.8, I51.4 |
| Myositis | M33, M33.0, M33.1, M33.2, M33.9, M60, M60.0, M60.00, M60.01, M60.02, M60.03, M60.04, M60.05, M60.07, M60.07, M60.08, M60.09, M60.1, M60.10, M60.11, M60.12, M60.13, M60.14, M60.15, M60.16, M60.17, M60.18, M60.19, M60.8, M60.80, M60.81, M60.82, M60.83, M60.84, M60.85, M60.86, M60.87, M60.88, M60.89, M60.9, M60.90, M60.91, M60.92, M60.93, M60.94, M60.95, M60.96, M06.97, M60.98, M60.99, M63, M63.0, M63.00, M63.01, M63.02, M63.03, M63.04, M63.05, M63.07, M63.07, M63.08, M63.09, M63.1,M63.10, M63.11, M63.12, M63.13, M63.14, M63.15, M63.16, M63.17, M63.18, M63.19, M63.2, M63.20, M63.21, M63.22, M63.23, M63.24, M63.25, M63.26, M63.27, M63.28, M63.29, M63.3, M63.30, M63.31, M63.32, M63.33, M63.34, M63.35, M63.36, M63.37, M63.38, M63.39 |
| Pericarditis | I01.0, I09.2, I30, I30.1, I30.8, I30.9, I31.0, I31.1, I32, I32.0, I32.1, I32.8 |
| Psoriatic arthritis | M07.0, M07.00, M07.04, M07.07, M07.09, M07.1, M07.10, M07.12, M07.13, M07.14, M07.15, M07.16, M07.17, M07.18, M07.19, M07.2, M07.3, M07.30, M07.31, M07.32, M07.33, M07.34, M07.35, M0.36, M07.37, M07.38, M07.39 |
| Peripheral vascular disease | W09.01, E09.02, E09.5, E09.51, E09.52, E10.51, E10.52, E11.51,E11.52, E13.51, E13.52, E14.51, E14.52, I70, I70.0, I70.1, I70.2,I70.20, I70.21, I70.22, I70.23, I70.24, I70.8, I70.9, I71, I71.0,I71.00, I71.01, I71.02, I71.03, I71.1, I71.2, I71.3, I71.4, I71.5,I71.6, I71.8, I71.9, I72, I72.0 I72.1, I72.2, I72.3, I72.4, I72.5,I72.6, I72.8, I72.9, I73, I73.0, I73.1, I73.8, I73.9, I74, I74.0,I74.1, I74.2, I74.3, I74.4, I74.5, I74.8, I74.9, I77, I77.0, I77.1,I77.2, I77.3, I77.4, I77.5, I77.6, I77.8, I77.9, I78, I78.0, I78.1,I78.8, I78.9, I79, I79.0, I79.1, I79.2, I79.8 |
| Rheumatoid arthritis | M05, M05.0, M05.00, M05.01, M05.02, M05.03, M05.04, M05.05, M05.06, M05.07, M05.08, M05.09, M05.1, M05.10, M05.11, M05.12, M05.13, M05.14, M05.15, M05.16, M05.17, M05.18, M05.19, M05.2, M05.20, M05.21, M05.22, M05.23, M05.24, M05.25, M05.26, M05.27, M05.28, M05.29, M05.3, M05.30, M05.31, M05.32, M05.33, M05.34, M05.35, M05.36, M05.37, M05.38, M05.39, M05.8, M05.80, M05.81, M05.82, M05.83, M05.84, M05.85, M05.86, M05.87, M05.88, M05.89, M05.9, M05.90, M05.91, M05.92, M05.93, M05.94, M05.95, M05.96, M05.97, M05.98, M05.99, M06, M06.0, M06.00, M06.01, M06.02, M06.03, M06.04, M06.05, M06.06, M06.07, M06.08, M06.09, M06.2, M06.20, M06.21, M06.22, M06.23, M06.24, M06.25, M06.26, M06.27, M06.28, M06.29, M06.3, M06.30, M06.31, M06.32, M06.33, M06.34, M06.35, M06.36, M06.37, M06.38, M06.39, M06.8, M06.80, M06.81, M06.82, M06.83, M06.84, M06.85, M06.86, M06.87, M06.88, M06.89, M06.9, M06.90, M06.91, M06.92, M06.93, M06.94, M06.95, M06.96, M06.97, M06.98, M06.99, U86.1 |
| Sarcoidosis | D86, D86.0, D86.1, D86.2, D86.3, D86.8, D86.9, G53.2, M63.3, M63.30, M63.31, M63.32, M63.33, M63.34, M63.35, M63.36, M63.37, M63.38, M63.39))#NB: there is no code for cardiac sarcoidosis of any form |
| Systemic lupus erythematosus | L93, L93.0, L93.1, L93.2, M32, M32.0, M32.1, M32.8, M32.9, U86.3 |
| Smoking | Z58.7, F17, F17.0, F17.2, F17.3, F17.4, F17.6, F17.7, F17.8, F17.9, T65.2, Z71.6, Z72.0, Z86.43 |
| Stroke | G46, G46.0, G46.1, G46.2, G46.3, G46.4, G46.5, G46.6, G46.7, G46.8, I63, I63.0, I63.1, I63.2, I63.3, I63.4, I63.5, I63.6, I63.8, I63.9, I69.3, I60, I60.0, I60.1, I60.2, I60.3, I60.4, I60.5, I60.6, I60.7, I60.8, I60.9, I61, I61.0, I61.1, I61.2, I61.3, I61.4, I61.5, I61.6, I61.8, I61.9, I62, I62.0, I62.1, I62.9, I64, I69.0, I69.1, I69.2, I64, I69.4 |
| Syncope | R55 |
| Transient ischaemic attack | G45, G45.0, G45.1, G45.2, G45.3, G45.4, G45.8, G45.9 |
| Ventricular arrhythmia | I47.0, I47.2, I49.0 |
| Venous thromboembolism | I26, I26.0, I26.9, I80, I80.0, I80.1, I80.2, I80.3, I80.8, I80.9, I81, I82, I82.0, I82.1, I82.2, I82.3, I82.8, I82.9 |
| Autoimmune disease | D86, D86.0, D86.1, D86.2, D86.3, D86.8, D86.9, G53.2, L93, L93.0, L93.1, L93.2, MO6, MO6, MO7, MO8, MO9, M30, M31, M32, M33, M34, M35, M36, M45, M46, M63.3, M63.30, M63.31, M63.32, M63.33, M63.34, M63.35, M63.36, M63.37, M63.38, M63.39, U86.3 |
| Malignancy | ICD-10 codes beginning with C |
| Respiratory disease | ICD-10 codes beginning with J, or Covid-19 |
| Digestive disease | ICD-10 codes beginning with K |
| Hip fracture | 47528-01, 49318-00, 49319-00 |

**Supplementary Table 3: Risk of myocarditis within 1-months of prespecified conditions**

| **Primary diagnosis for hospitalisations in the 1-month preceding index myocarditis admission** | **Odds ratio (95% CI)** | **p-value** |
| --- | --- | --- |
| Myocardial infarction | 134.3 (63.9-282.3) | <0.0001 |
| Pericarditis | 106.5 (22.7-500.3) | <0.0001 |
| Heart failure | 30.5 (16.2-57.1) | <0.0001 |
| Influenza | 32 (4.4-233.4) | <0.0001 |
| Ventricular arrhythmia | 33.8 (7.4-154.6) | <0.0001 |
| Covid-19 | 64.8 (15.5-270.5) | <0.0001 |
| Respiratory disease | 17.3 (11.1-26.9) | <0.0001 |
| Autoimmune disease | 16.5 (7.4-36.9) | <0.0001 |
| Atrial fibrillation | 15.9 (6.3-40.2) | <0.0001 |
| Myositis | 22.8 (2.9-180.9) | <0.0001 |
| Sarcoidosis | NA | NA |
| Stroke | 3.3 (0.5-22.1) | 0.0687 |
| Systemic lupus erythematosus | 11.3 (0.7-190.1) | 0.0119 |
| Digestive disease | 3.5 (2.2-5.8) | <0.0001 |
| Diabetes | 1.5 (0.2-14.9) | 0.58 |
| Malignancy | 1.3 (0.5-3.4) | 0.4439 |

Odds of risk factors up to 30-days prior to myocarditis admission compared to 360-720 days prior to myocarditis admission using conditional logistic regression
Conditions required to be primary diagnosis for the hospital admission, except for Covid-19 which was based on secondary diagnosis
NA indicates unbounded estimate due to small numbers

**Supplementary Table 4: Risk of myocarditis within 3-months of prespecified conditions**

| **Primary diagnosis for hospitalisations in the 3-months preceding index myocarditis admission** | **Odds ratio (95% CI)** | **p-value** |
| --- | --- | --- |
| Myocardial infarction | 1.8 (0.5-6) | 0.1654 |
| Pericarditis | 1.7 (0.1-22.2) | 0.5602 |
| Heart failure | 1.6 (0.6-4) | 0.1462 |
| Influenza | 1.3 (0-39.5) | 0.8033 |
| Ventricular arrhythmia | 0.6 (0-13.7) | 0.589 |
| Covid-19 | 10.5 (1.5-75.8) | 0.0005 |
| Respiratory disease | 2.0 (1.1-3.6) | 0.0004 |
| Autoimmune disease | 2.7 (1-7.4) | 0.005 |
| Atrial fibrillation | 2.4 (0.8-7.4) | 0.0178 |
| Myositis | 5.4 (0.1-221.4) | 0.1795 |
| Sarcoidosis | NA | NA |
| Stroke | 0.4 (0-7.8) | 0.323 |
| Systemic lupus erythematosus | 5.2 (0.2-174.6) | 0.1705 |
| Digestive disease | 1.2 (0.7-1.9) | 0.3971 |
| Diabetes | 0.7 (0.1-5.4) | 0.6069 |
| Malignancy | 0.8 (0.4-1.8) | 0.4234 |

Odds of risk factors up to 90-days prior to myocarditis admission compared to 91-450 days prior to myocarditis admission using conditional logistic regression
Conditions required to be primary diagnosis for the hospital admission, except for Covid-19 which was based on secondary diagnosis
NA indicates unbounded estimate due to small numbers

**Supplementary Table 5: Risk of myocarditis 90-120 days after prespecified conditions**

| **Primary diagnosis for hospitalisations in the 90-120 days preceding index myocarditis admission** | **Odds ratio (95% CI)** | **p-value** |
| --- | --- | --- |
| Myocardial infarction | 2.6 (0.7-9) | 0.0273 |
| Pericarditis | 3.7 (0.5-26.5) | 0.0506 |
| Heart failure | 4.4 (1.9-10.2) | <0.0001 |
| Influenza | NA | NA |
| Ventricular arrhythmia | 2.9 (0.3-31.6) | 0.1999 |
| Covid-19 | 3.2 (0.7-14.2) | 0.0218 |
| Respiratory disease | 1.3 (0.6-2.9) | 0.3183 |
| Autoimmune disease | 1.4 (0.5-4.3) | 0.3245 |
| Atrial fibrillation | 1 (0.2-5) | 1 |
| Myositis | 2.8 (0.3-25.6) | 0.1802 |
| Sarcoidosis | NA | NA |
| Stroke | 3.6 (0.3-40) | 0.1218 |
| Systemic lupus erythematosus | 2.5 (0.2-31.4) | 0.2821 |
| Digestive disease | 1.1 (0.5-2.3) | 0.6876 |
| Diabetes | NA | NA |
| Malignancy | 2.4 (1-5.3) | 0.002 |

Odds of risk factors 90-120 days prior to myocarditis admission compared to 121-480 days prior to myocarditis admission using conditional logistic regression
Conditions required to be primary diagnosis for the hospital admission, except for Covid-19 which was based on secondary diagnosis
NA indicates unbounded estimate due to small numbers

**Supplementary Table 6: Summary features of study cohort during index pericarditis admission**

| **Feature** | **Value** |
| --- | --- |
| Male | 68.5% (9794/14290) |
| Age (years) | 56.7 (37-70.5) |
| Private hospital | 16.4% (2338/14290) |
| Major city^a^ | 71.6% (10217/14279) |
| Rural^b^ | 5.3% (756/14279) |
| Heart failure | 16.7% (2387/14290) |
| Ischemic heart disease | 13.2% (1881/14290) |
| Atrial fibrillation | 26.2% (3751/14290) |
| Diabetes | 16% (2281/14290) |
| Malignancy | 13.7% (1957/14290) |
| Ventricular arrhythmia | 4.3% (616/14290) |
| Autoimmunity | 5.8% (826/14290) |
| Chronic kidney disease | 8.5% (1220/14290) |
| Pulmonary hypertension | 4.5% (641/14290) |
| Charlson comorbidity index | 2 (0-5) |

Continuous variables reported as median (interquartile [IQR]). Binary variables reported as percentage (frequency).
a. Major city defined by Australian Statistical Geography Standard of 1

b. Rural defined by Australian Statistical Geography Standard of 3 or more

**Supplementary Table 7: Summary features of study cohort during index hip fracture repair admission**

| **Feature** | **Value** |
| --- | --- |
| Male | 45.5% (61635/135567) |
| Age (years) | 69.4 (60.6-77.3) |
| Private hospital | 55.3% (74949/135567) |
| Major city^a^ | 65.9% (89245/135522) |
| Rural^b^ | 6.5% (8766/135522) |
| Heart failure | 4.4% (5905/135567) |
| Ischemic heart disease | 4% (5446/135567) |
| Atrial fibrillation | 9.7% (13165/135567) |
| Diabetes | 13.3% (18038/135567) |
| Malignancy | 15.5% (21035/135567) |
| Ventricular arrhythmia | 0.8% (1022/135567) |
| Autoimmunity | 4% (5362/135567) |
| Chronic kidney disease | 3.6% (4862/135567) |
| Pulmonary hypertension | 0.8% (1086/135567) |
| Charlson comorbidity index | 3 (2-5) |

Continuous variables reported as median (interquartile [IQR]). Binary variables reported as percentage (frequency).
a. Major city defined by Australian Statistical Geography Standard of 1

b. Rural defined by Australian Statistical Geography Standard of 3 or more

**Supplementary Table 8: Risk of pericarditis (positive control) within 1-month of prespecified conditions**

| **Primary diagnosis for hospitalisations in the 1-month preceding index pericarditis admission** | **Odds ratio (95% CI)** | **p-value** |
| --- | --- | --- |
| Myocarditis | 37.6 (11.4-123.4) | <0.0001 |
| Myocardial infarction | 31.2 (24.7-39.3) | <0.0001 |
| Heart failure | 6.1 (4.7-8) | <0.0001 |
| Influenza | 2.7 (0.8-9.3) | 0.0172 |
| Ventricular arrhythmia | 6.6 (2.9-15.3) | <0.0001 |
| Covid-19 | 13.8 (8.7-22) | <0.0001 |
| Respiratory disease | 6.2 (5.2-7.4) | <0.0001 |
| Autoimmune disease | 5.3 (3.6-7.8) | <0.0001 |
| Atrial fibrillation | 5.7 (4.2-7.7) | <0.0001 |
| Myositis | 6.8 (0.5-102.5) | 0.038 |
| Sarcoidosis | NA | NA |
| Stroke | 2.4 (1-5.6) | 0.0033 |
| Systemic lupus erythematosus | 3.1 (0.9-10.4) | 0.0071 |
| Digestive disease | 1.4 (1.1-1.8) | 0.0002 |
| Diabetes | 1.6 (0.8-3.2) | 0.0488 |
| Malignancy | 2.5 (1.8-3.3) | <0.0001 |

Odds of risk factors up to 30 days prior to pericarditis admission compared to 31-390 days prior to pericarditis admission using conditional logistic regression
Conditions required to be primary diagnosis for the hospital admission, except for Covid-19 which was based on secondary diagnosis
NA indicates unbounded estimate due to small numbers
Number of unique patients with index admission for pericarditis: 14,290

**Supplementary Table 9: Risk of hip fracture repair (negative control) within 1-month of prespecified conditions**

| **Primary diagnosis for hospitalisations in the 1-month preceding index hip fracture repair** | **Odds ratio (95% CI)** | **p-value** |
| --- | --- | --- |
| Myocarditis | NA | NA |
| Myocardial infarction | 0.4 (0.2-0.8) | <0.0001 |
| Pericarditis | 1.3 (0.1-30.6) | 0.7832 |
| Heart failure | 0.9 (0.6-1.4) | 0.5141 |
| Influenza | 0.5 (0.1-2.7) | 0.2121 |
| Ventricular arrhythmia | 0.9 (0.2-4) | 0.7633 |
| Covid-19 | 1.8 (1-3.3) | 0.0037 |
| Respiratory disease | 0.7 (0.6-0.9) | <0.0001 |
| Autoimmune disease | 1.2 (1-1.6) | 0.016 |
| Atrial fibrillation | 0.9 (0.6-1.2) | 0.2669 |
| Myositis | 1 (0.3-4) | 0.9434 |
| Sarcoidosis | NA | NA |
| Stroke | 0.7 (0.4-1.2) | 0.058 |
| Systemic lupus erythematosus | 0.8 (0.1-7.8) | 0.8265 |
| COPD | 0.6 (0.4-0.9) | 5.00E-04 |
| Digestive | 0.7 (0.7-0.8) | <0.0001 |
| Diabetes | 0.9 (0.6-1.4) | 0.4941 |
| Malignancy | 1.1 (0.9-1.2) | 0.2003 |

Odds of risk factors up to 30 days prior to hip fracture repair compared to 31-390 days prior to hip fracture repair using conditional logistic regression
Conditions required to be primary diagnosis for the hospital admission, except for Covid-19 which was based on secondary diagnosis
NA indicates unbounded estimate due to small numbers
Number of unique patients with index admission for hip fracture repair: 135,567

**Supplementary Table 10: Predictors of myocarditis recurrence using different methodology: Cox regression, competing risk analysis according to Fine and Gray, adjustment and without adjustment**

| **Variables** | **Total count (eg. number of males in cohort)** | **Total count with recurrent myocarditis (eg. number of males in cohort who have recurrent myocarditis)** | **Adjusted hazard ratio (Cause-specific Cox regression)** | **p-value** | **Unadjusted hazard ratio (Cause-specific Cox regression)** | **p-value** | **Adjusted subdistribution hazard ratio (Fine and Gray)** | **p-value** | **Unadjusted subdistribution hazard ratio (Fine and Gray)** | **p-value** |
| --- | --- | --- | --- | --- | --- | --- | --- | --- | --- | --- |
|  |  |  |  |  |  |  |  |  |  |  |
| **Demographics** **at time of index myocarditis admission** | | | | | | | | | | |
| Sex (male) | 2690 | 83 | 0.93 (0.63-1.36) | 0.704 | 1 (0.69-1.46) | 0.984 | 0.93 (0.64-1.36) | 0.7 | 1.03 (0.71-1.49) | 0.89 |
| Age (per 10 years) | 4071 | 124 | 0.83 (0.74-0.93) | 0.001 | 0.88 (0.8-0.96) | 0.003 | 0.84 (0.75-0.94) | 0.002 | 0.86 (0.79-0.93) | <0.001 |
| **Hospitalisation characteristics** **at time of index myocarditis admission** | | | | | | | | | | |
| Admission duration (weeks) | 4071 | 124 | 1 (0.97-1.02) | 0.702 | 1 (0.97-1.02) | 0.697 | 1 (0.97-1.02) | 0.71 | 1 (0.97-1.02) | 0.71 |
| Admitted to private hospital | 333 | 3 | 0.3 (0.1-0.97) | 0.044 | 0.26 (0.08-0.82) | 0.022 | 0.32 (0.1-1.02) | 0.054 | 0.27 (0.08-0.84) | 0.024 |
| Intensive care unit admission | 625 | 13 | 0.6 (0.33-1.1) | 0.1 | 0.74 (0.42-1.31) | 0.301 | 0.54 (0.29-1) | 0.052 | 0.64 (0.36-1.13) | 0.12 |
| Hospital in major city^a^ | 3007 | 91 | 0.95 (0.64-1.41) | 0.792 | 0.97 (0.65-1.44) | 0.862 | 0.95 (0.64-1.42) | 0.81 | 0.97 (0.65-1.44) | 0.88 |
| Hospital in rural area^b^ | 235 | 6 | 0.84 (0.37-1.9) | 0.673 | 0.82 (0.36-1.87) | 0.644 | 0.85 (0.37-1.92) | 0.69 | 0.83 (0.37-1.87) | 0.65 |
| **Comorbidities at time of index myocarditis admission** | | | | | | | | | | |
| Charlson comorbidity index | 4071 | 124 | 1.07 (0.96-1.19) | 0.208 | 0.96 (0.88-1.05) | 0.373 | 1.03 (0.92-1.15) | 0.62 | 0.93 (0.85-1.01) | 0.08 |
| Idiopathic myocarditis^c^ | 2317 | 74 | 0.94 (0.64-1.37) | 0.744 | 0.97 (0.67-1.39) | 0.855 | 0.97 (0.66-1.42) | 0.88 | 1.07 (0.75-1.53) | 0.71 |
| Digestive disease | 1732 | 55 | 1.38 (0.93-2.04) | 0.105 | 1.22 (0.86-1.73) | 0.266 | 1.37 (0.93-2.02) | 0.11 | 1.13 (0.8-1.61) | 0.49 |
| Respiratory disease | 1695 | 44 | 0.85 (0.56-1.27) | 0.414 | 0.88 (0.61-1.27) | 0.494 | 0.83 (0.55-1.24) | 0.36 | 0.81 (0.56-1.17) | 0.26 |
| Reactive myocarditis^d^ | 1537 | 44 | 1.04 (0.71-1.53) | 0.849 | 1.04 (0.72-1.5) | 0.844 | 1.00 (0.68-1.47) | 0.99 | 0.95 (0.66-1.37) | 0.78 |
| Heart failure | 955 | 33 | 1.54 (1.02-2.32) | 0.038 | 1.33 (0.89-1.98) | 0.161 | 1.40 (0.93-2.12) | 0.10 | 1.17 (0.79-1.75) | 0.43 |
| Myocardial infarction | 640 | 21 | 1.5 (0.93-2.41) | 0.098 | 1.18 (0.74-1.89) | 0.487 | 1.48 (0.91-2.39) | 0.11 | 1.11 (0.7-1.78) | 0.66 |
| Atrial fibrillation | 507 | 13 | 1.21 (0.62-2.37) | 0.575 | 0.94 (0.53-1.66) | 0.82 | 1.16 (0.59-2.28) | 0.66 | 0.82 (0.46-1.45) | 0.49 |
| Diabetes | 423 | 5 | 0.5 (0.2-1.22) | 0.126 | 0.41 (0.17-0.99) | 0.048 | 0.47 (0.19-1.15) | 0.098 | 0.37 (0.15-0.9) | 0.028 |
| Malignancy | 321 | 10 | 2 (0.99-4.06) | 0.054 | 1.37 (0.72-2.62) | 0.343 | 1.64 (0.81-3.31) | 0.17 | 1.06 (0.56-2.03) | 0.86 |
| Covid-19 | 301 | 5 | 0.69 (0.29-1.68) | 0.419 | 0.72 (0.29-1.75) | 0.463 | 0.71 (0.29-1.73) | 0.45 | 0.7 (0.29-1.72) | 0.44 |
| Ventricular arrhythmia | 253 | 12 | 1.96 (1.04-3.66) | 0.036 | 1.86 (1.03-3.37) | 0.041 | 1.87 (1.00-3.49) | 0.051 | 1.66 (0.92-3.01) | 0.093 |
| Autoimmune disease | 229 | 9 | 1.47 (0.74-2.92) | 0.277 | 1.39 (0.71-2.72) | 0.344 | 1.52 (0.76-3.03) | 0.23 | 1.33 (0.68-2.61) | 0.41 |
| Pericarditis | 227 | 7 | 0.93 (0.43-2) | 0.852 | 0.99 (0.46-2.11) | 0.974 | 0.92 (0.43-1.98) | 0.84 | 1 (0.47-2.14) | 1 |
| Influenza | 136 | 3 | 0.8 (0.25-2.54) | 0.705 | 0.79 (0.25-2.49) | 0.687 | 0.80 (0.25-2.55) | 0.71 | 0.74 (0.24-2.33) | 0.61 |
| Stroke | 89 | 2 | 1.16 (0.28-4.76) | 0.833 | 0.91 (0.22-3.67) | 0.893 | 1.01 (0.25-4.14) | 0.99 | 0.76 (0.19-3.06) | 0.7 |
| Myositis | 62 | 2 | 1.13 (0.28-4.6) | 0.869 | 1.11 (0.28-4.44) | 0.884 | 1.16 (0.28-4.73) | 0.84 | 1.05 (0.26-4.21) | 0.94 |
| Systemic lupus erythematosus | 50 | 1 | 0.57 (0.08-4.11) | 0.581 | 0.64 (0.09-4.53) | 0.659 | 0.61 (0.08-4.35) | 0.62 | 0.64 (0.09-4.5) | 0.65 |
| Sarcoidosis | 32 | 2 | 2.45 (0.61-9.78) | 0.204 | 2.1 (0.53-8.4) | 0.293 | 2.65 (0.66-10.59) | 0.17 | 2.19 (0.55-8.76) | 0.27 |

Adjusted analyses were adjusted for age, sex and Charlson comorbidity index.

1. Australian Statistical Geography Standard 1
2. Australian Statistical Geography Standard ≥3
3. Idiopathic myocarditis defined as the absence of reactive myocarditis or the comorbidities autoimmune disease or malignancy
4. Reactive myocarditis defined as having respiratory or digestive system illness at presentation or requiring hospitalisation in the past 30-days

**Supplementary Table 11: Variance inflation factors of Cox multivariable regression model**

| **Covariate** | **Variance inflation factor** |
| --- | --- |
| Index admission at private hospital | 1.057 |
| Age (10 years) | 1.130 |
| Heart failure at index admission | 1.140 |
| Hospitalisation for autoimmune disease | 1.101 |
| Hospitalisation for Covid-19 | 1.147 |
| Hospitalisation for ventricular arrhythmia | 1.058 |
| Hospitalisation for pericarditis | 1.067 |

Variance inflation factors reported on model in Figure 3

**Supplementary Figure 1: Grouping of cases and controls for conditional logistic regression**

Myocarditis

Case

1 months

3 months

5 months

7 months

9 months

2 months

4 months

6 months

8 months

10 months

11 months

12 months

Controls

(a)

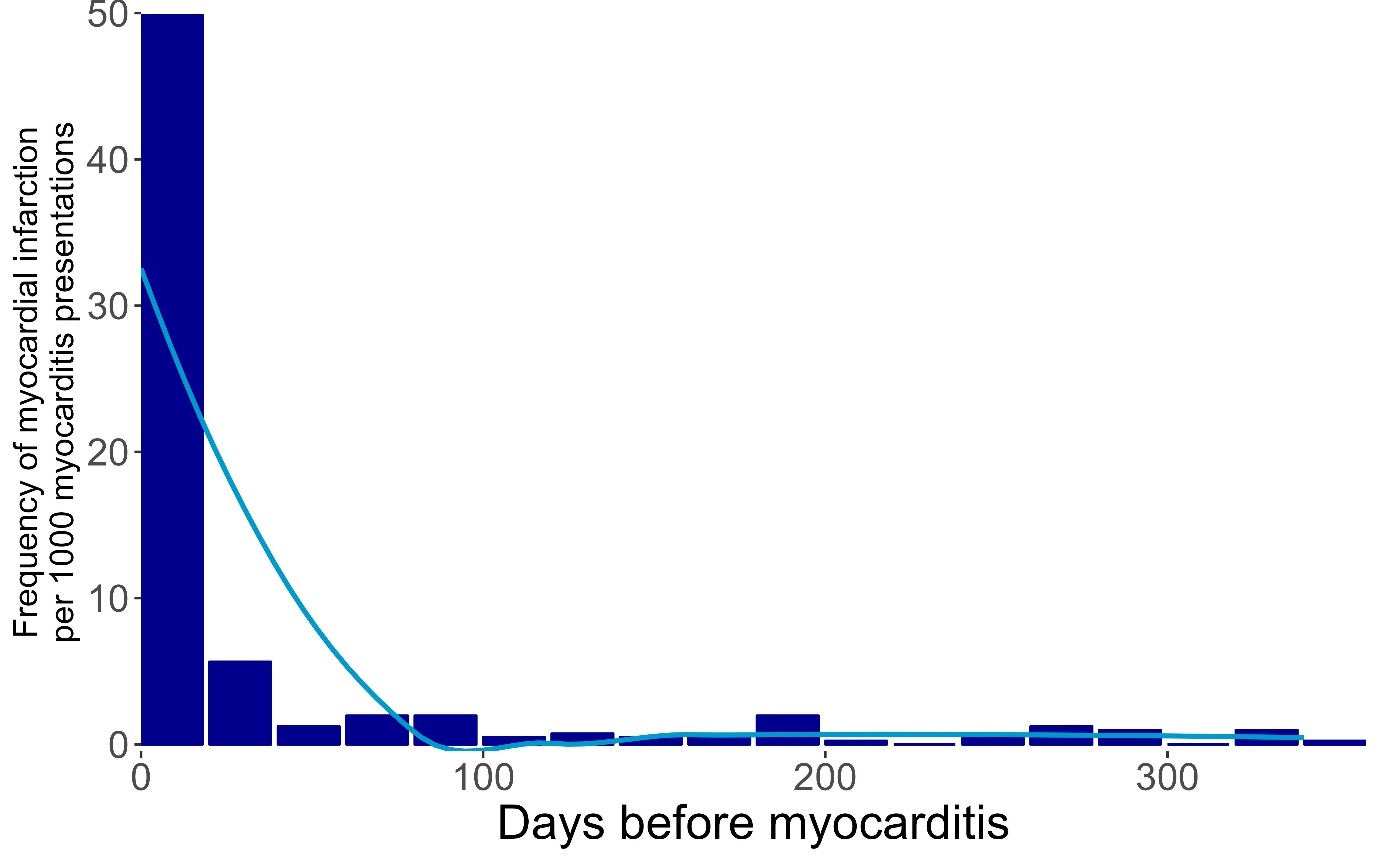


(b)

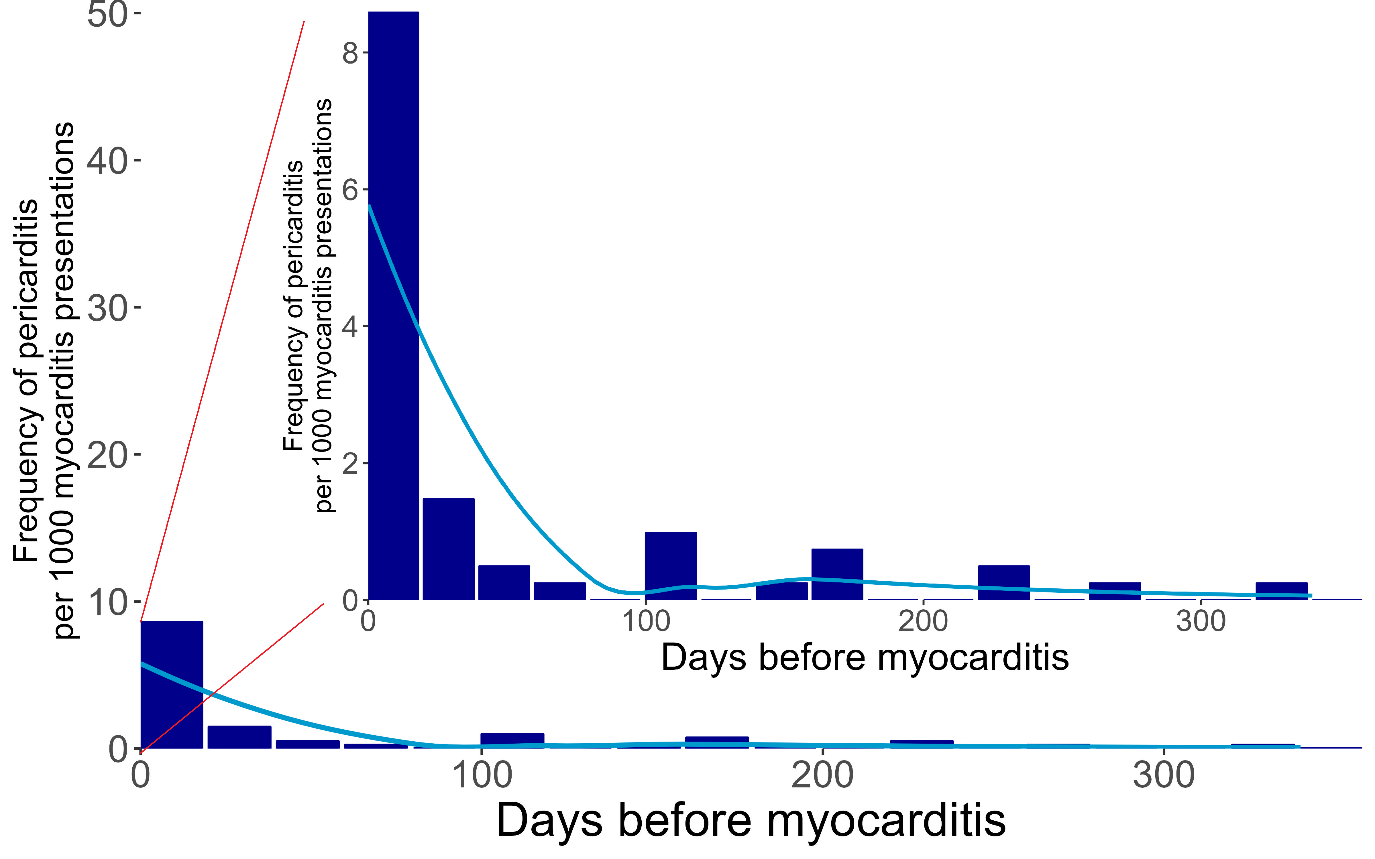


(c)

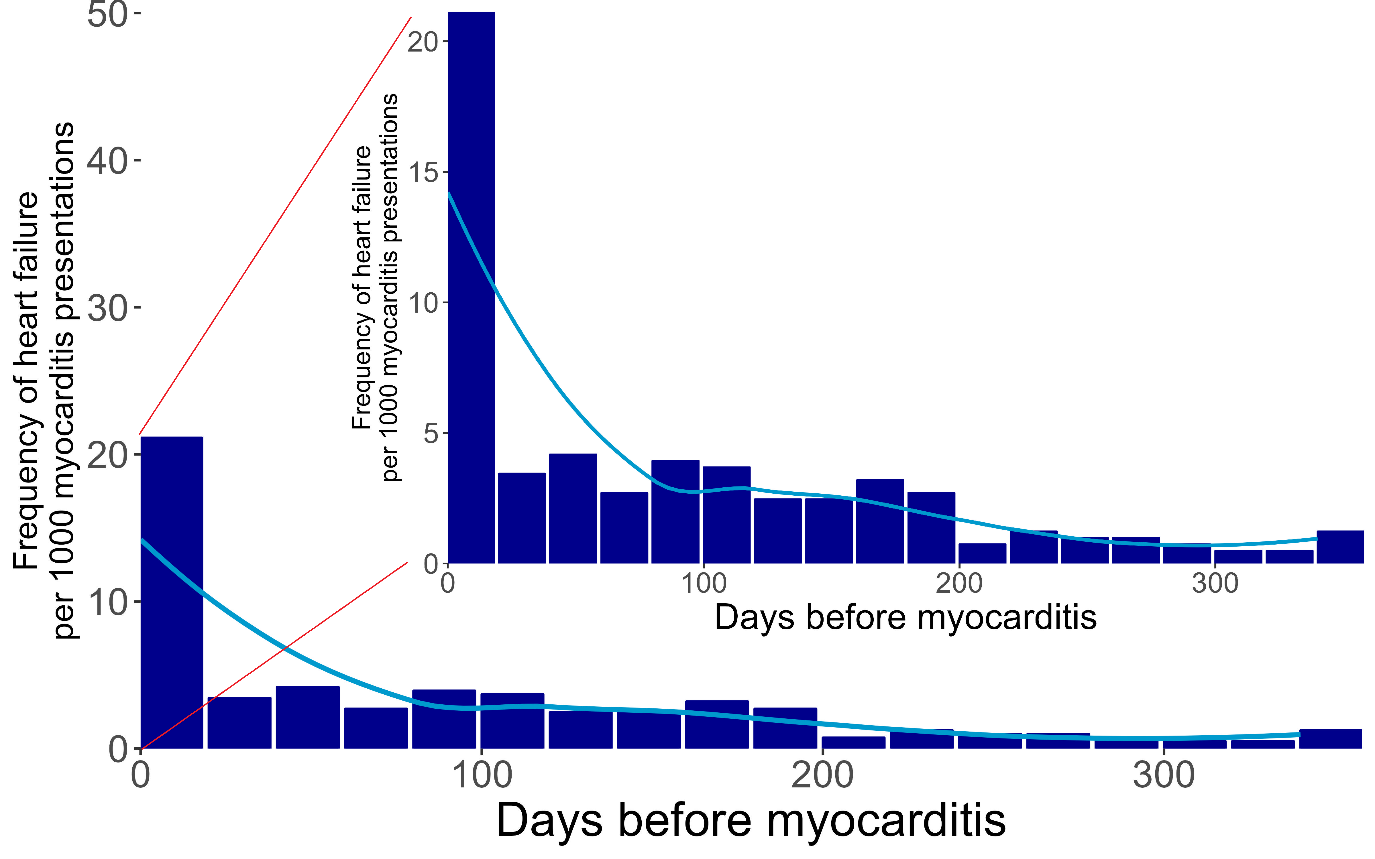


(d)

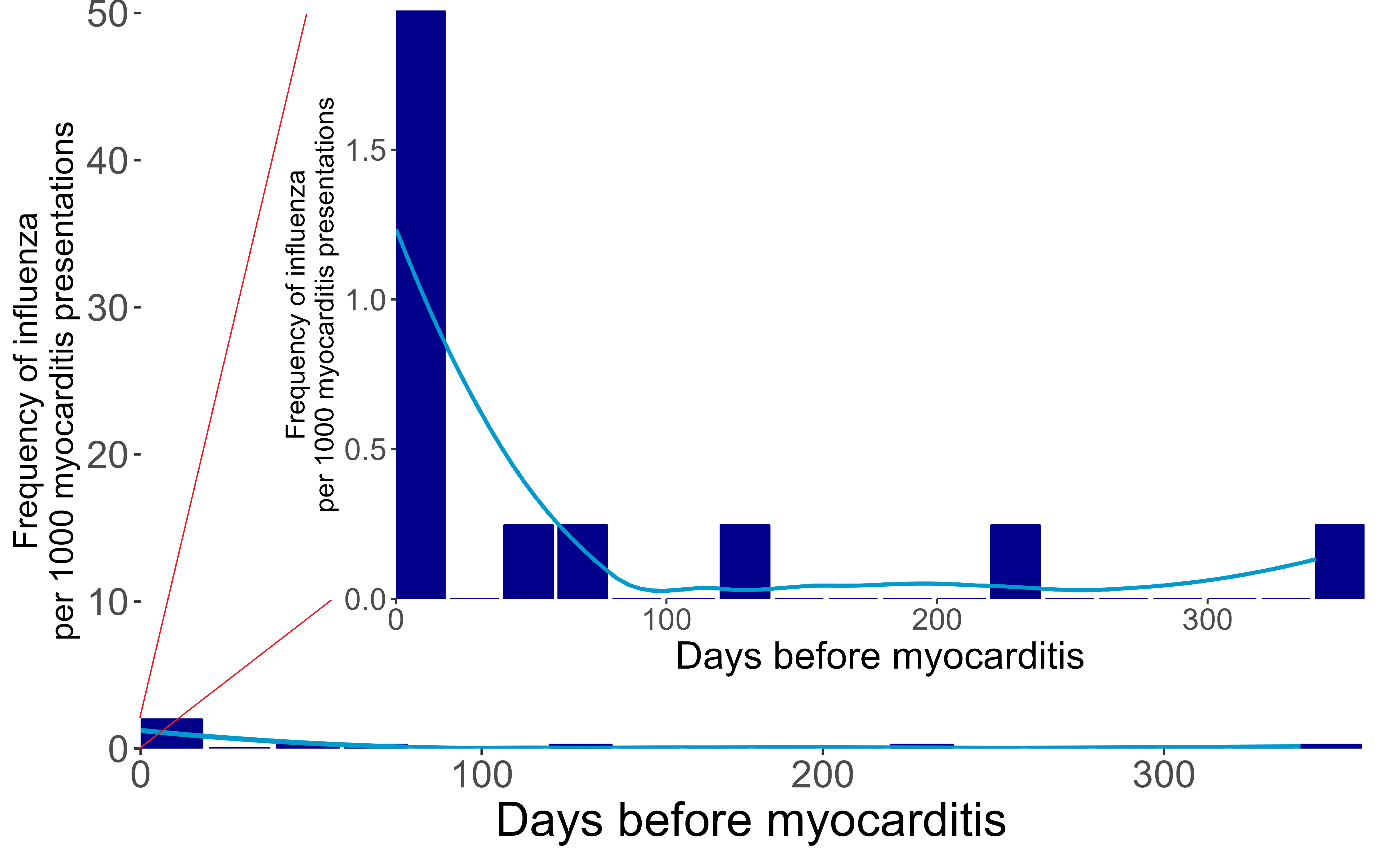


(e)

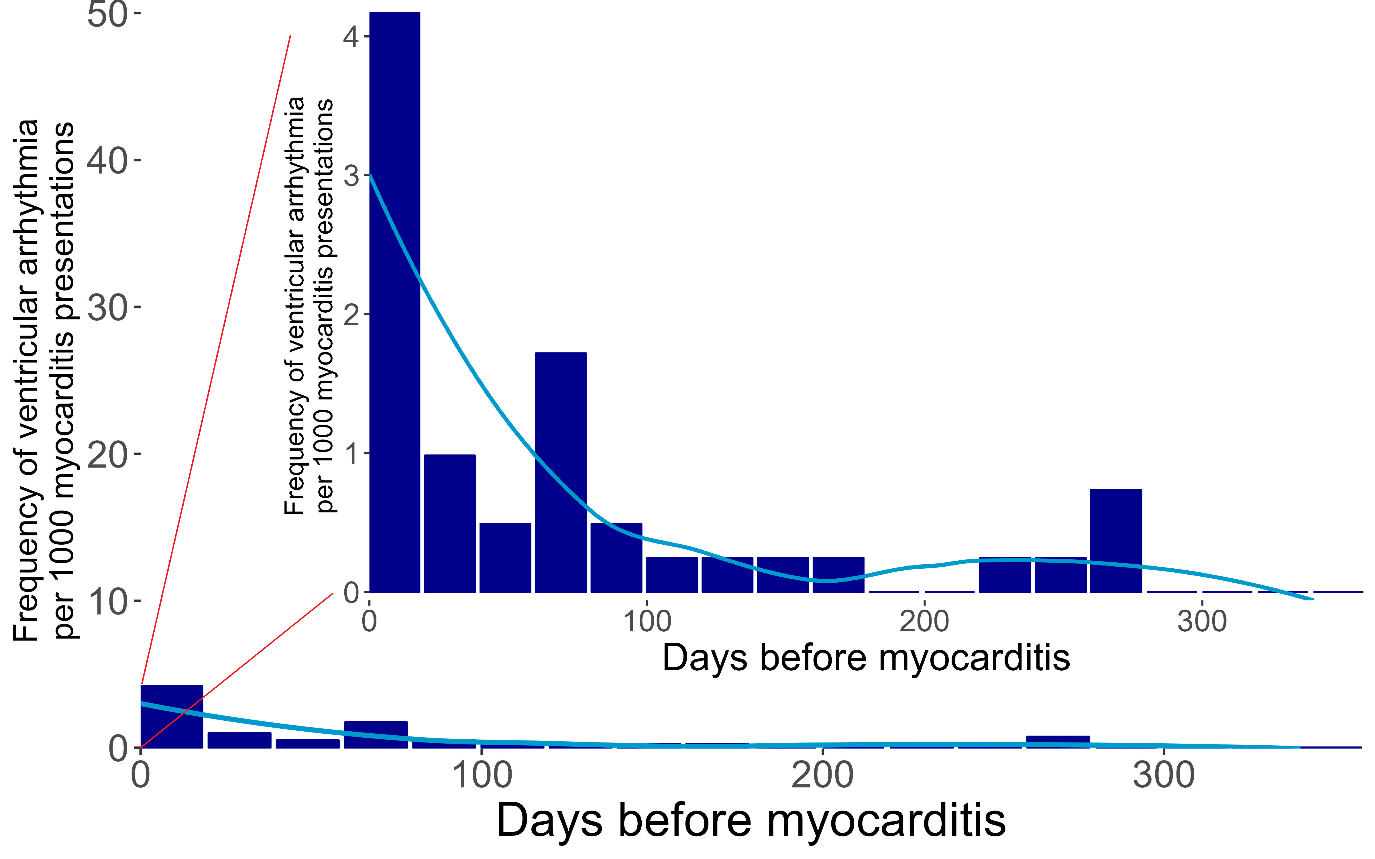


(f)

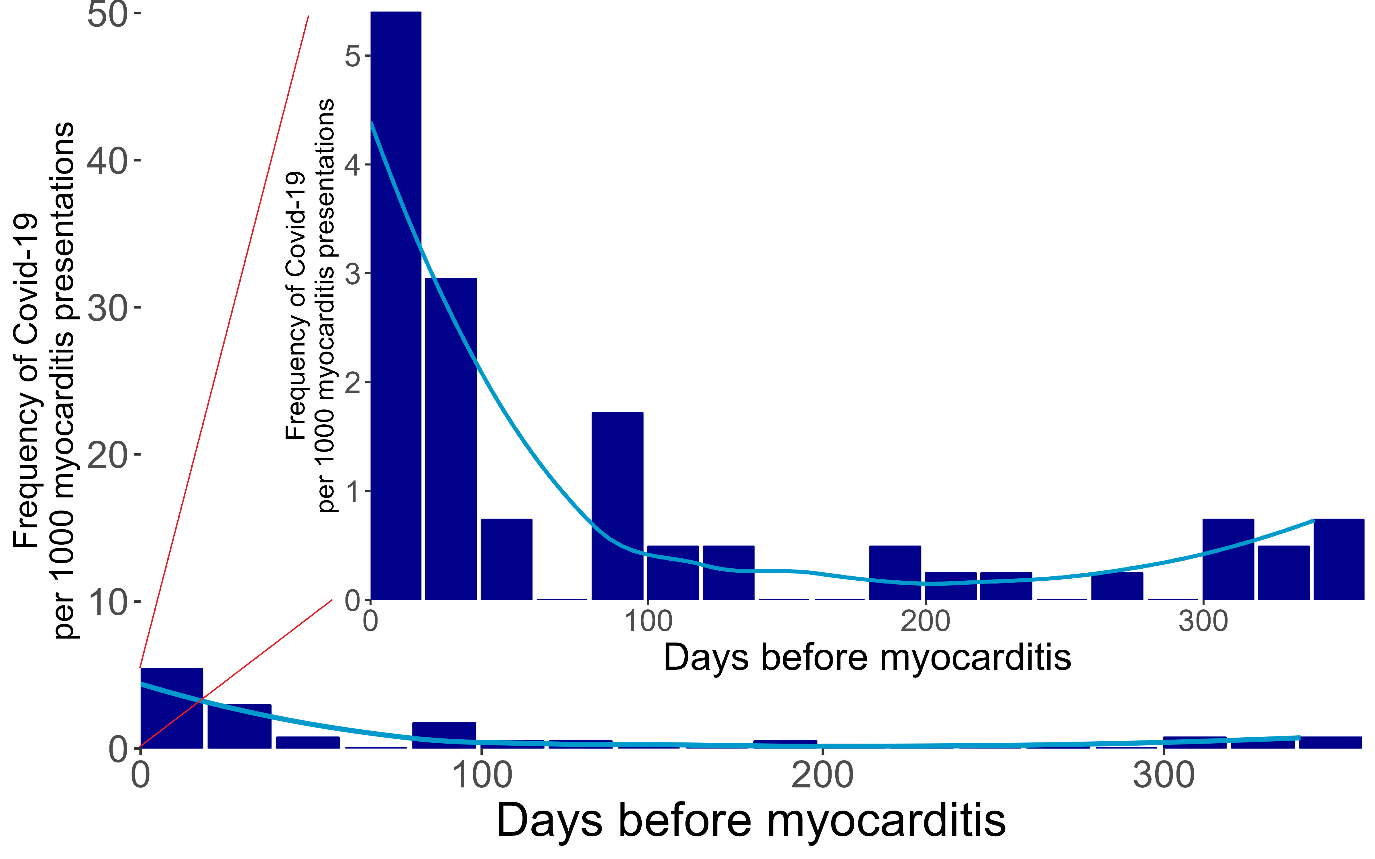


(g)

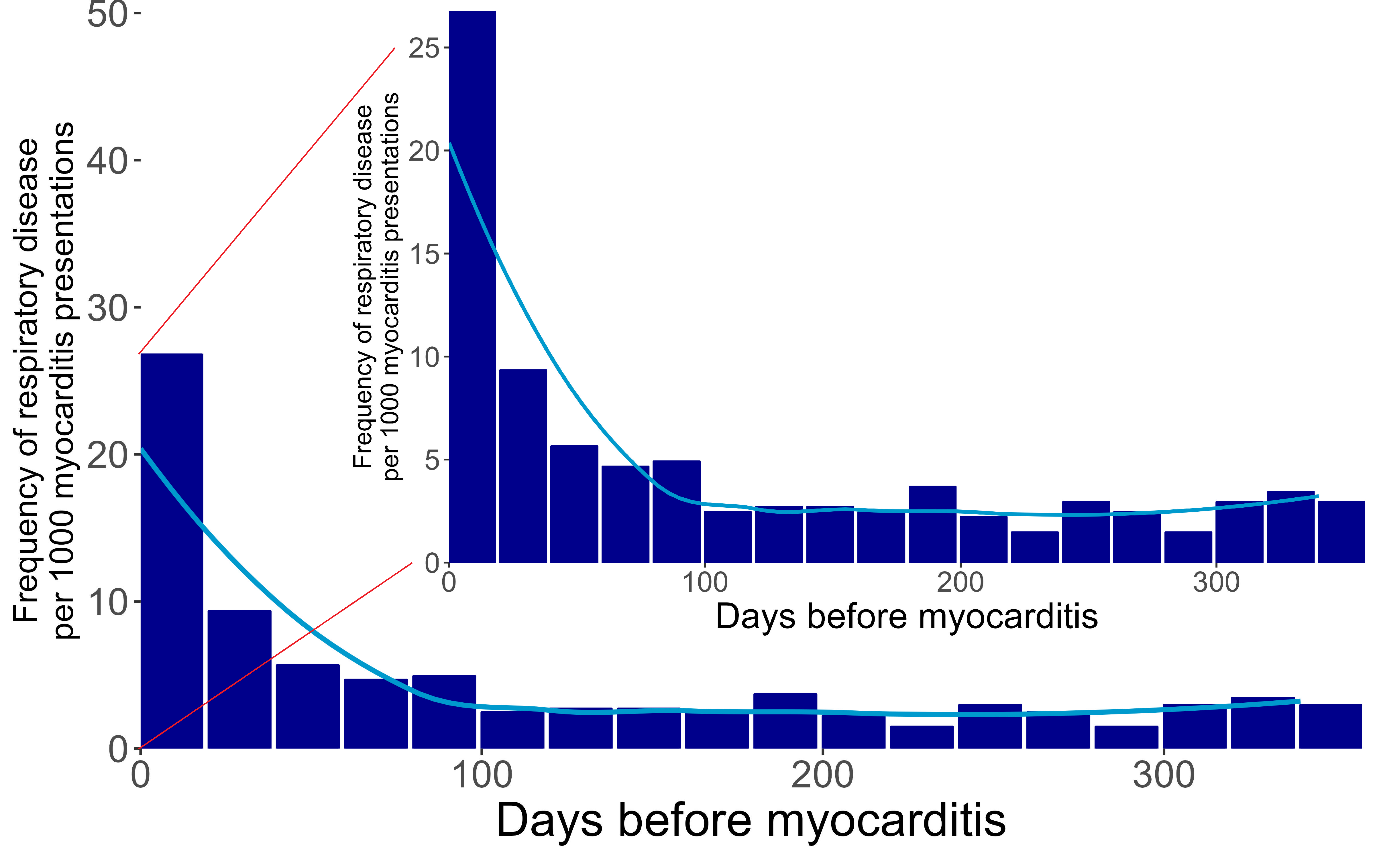


(h)

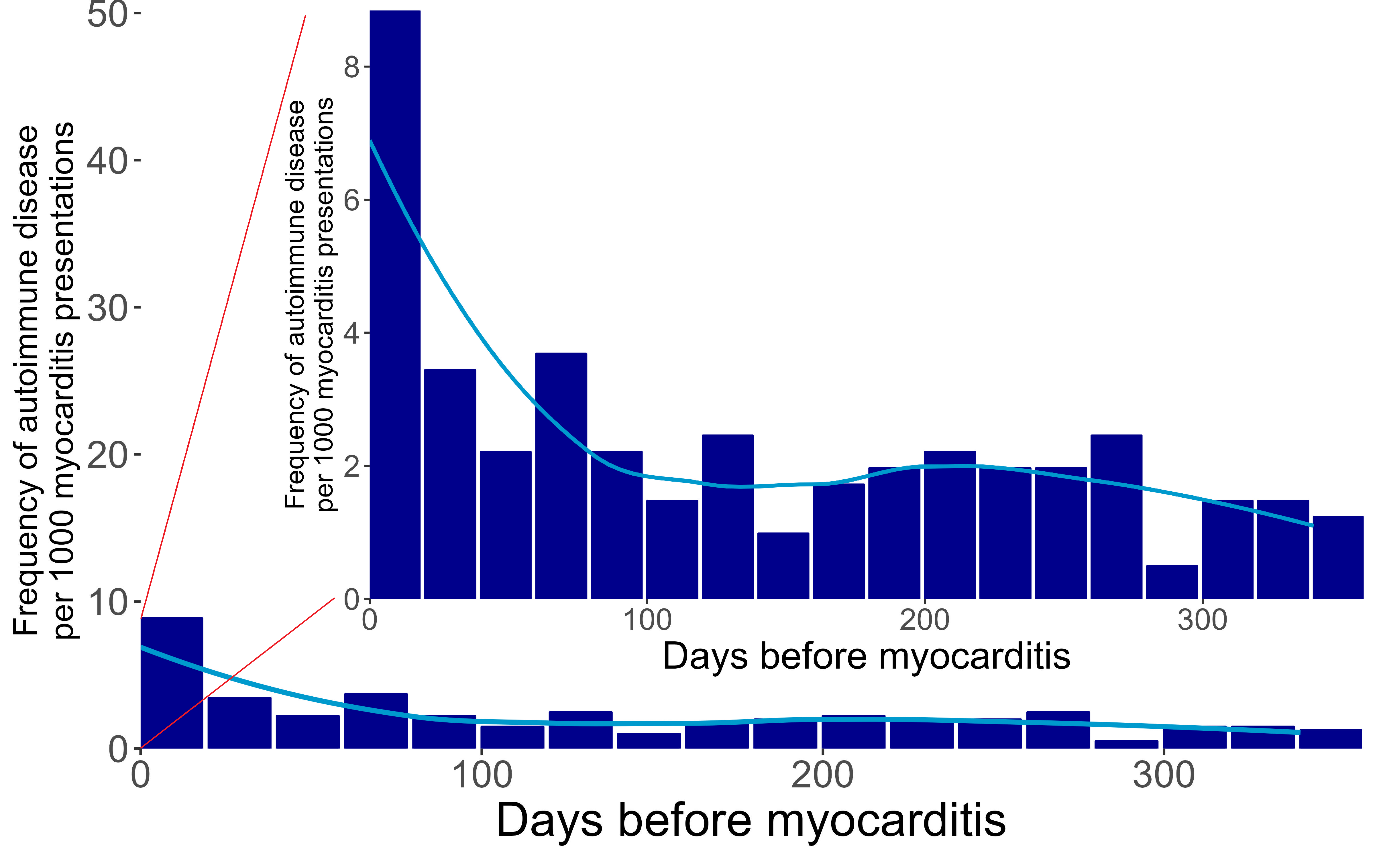


(i)

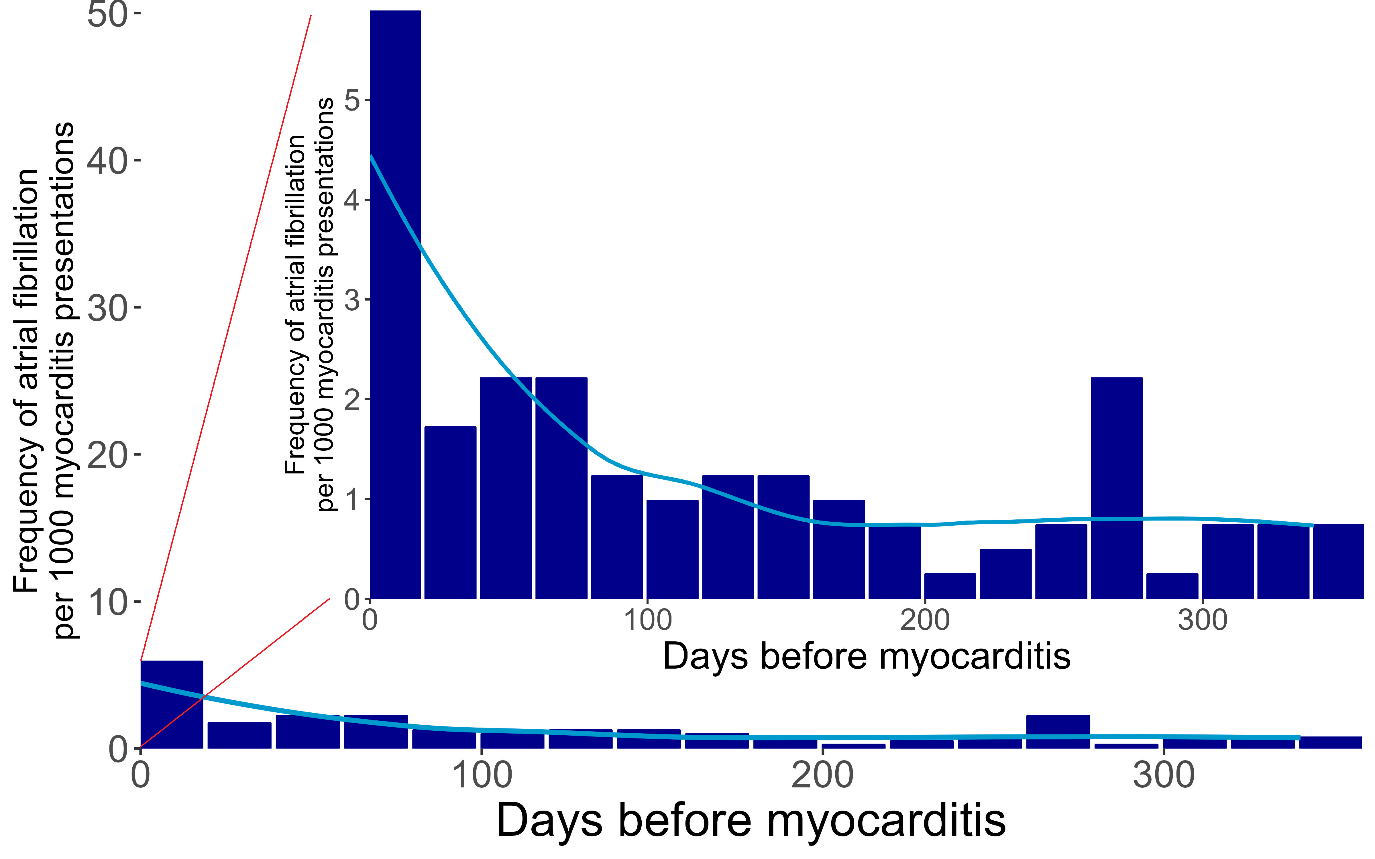


(j)

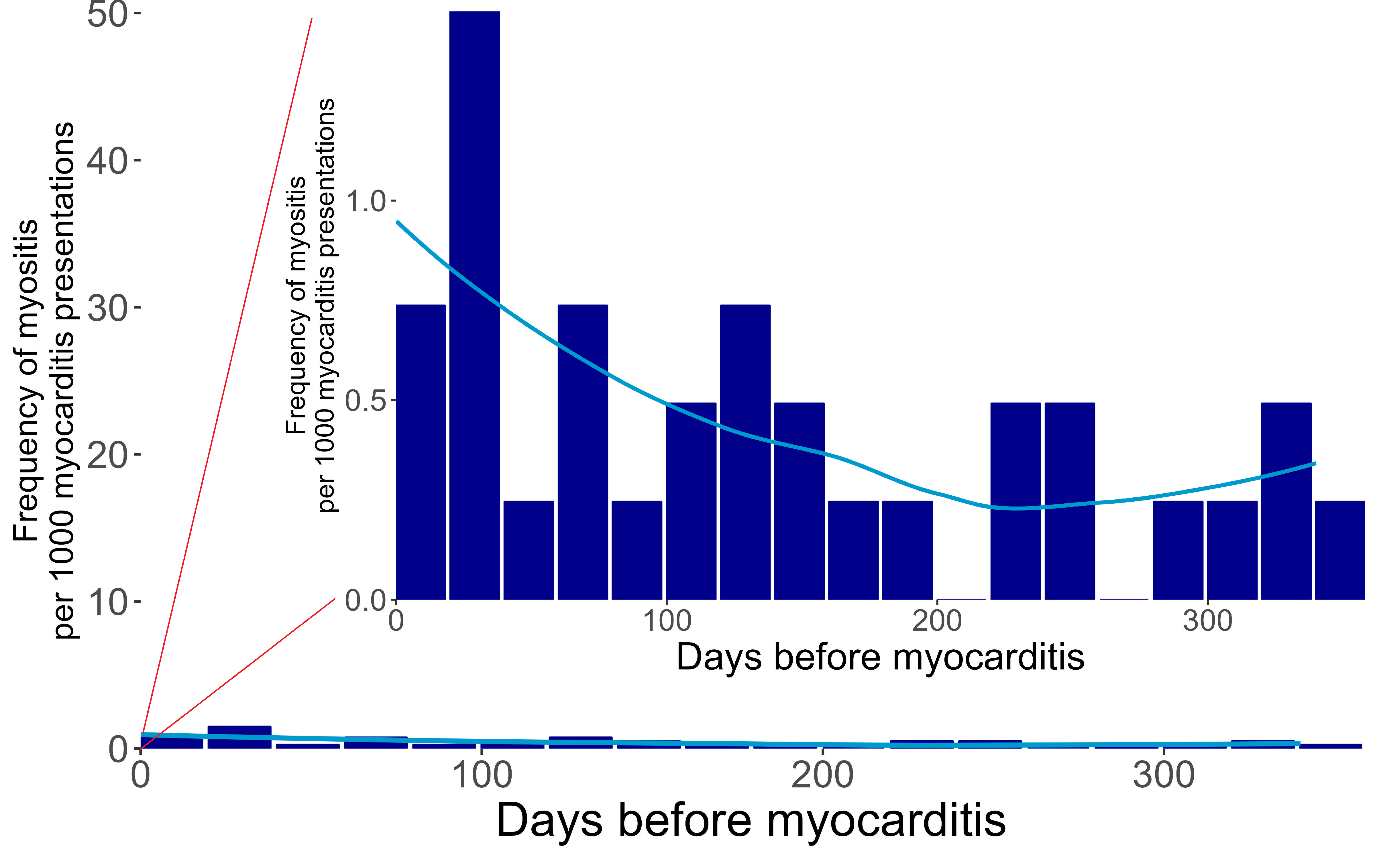


(k)

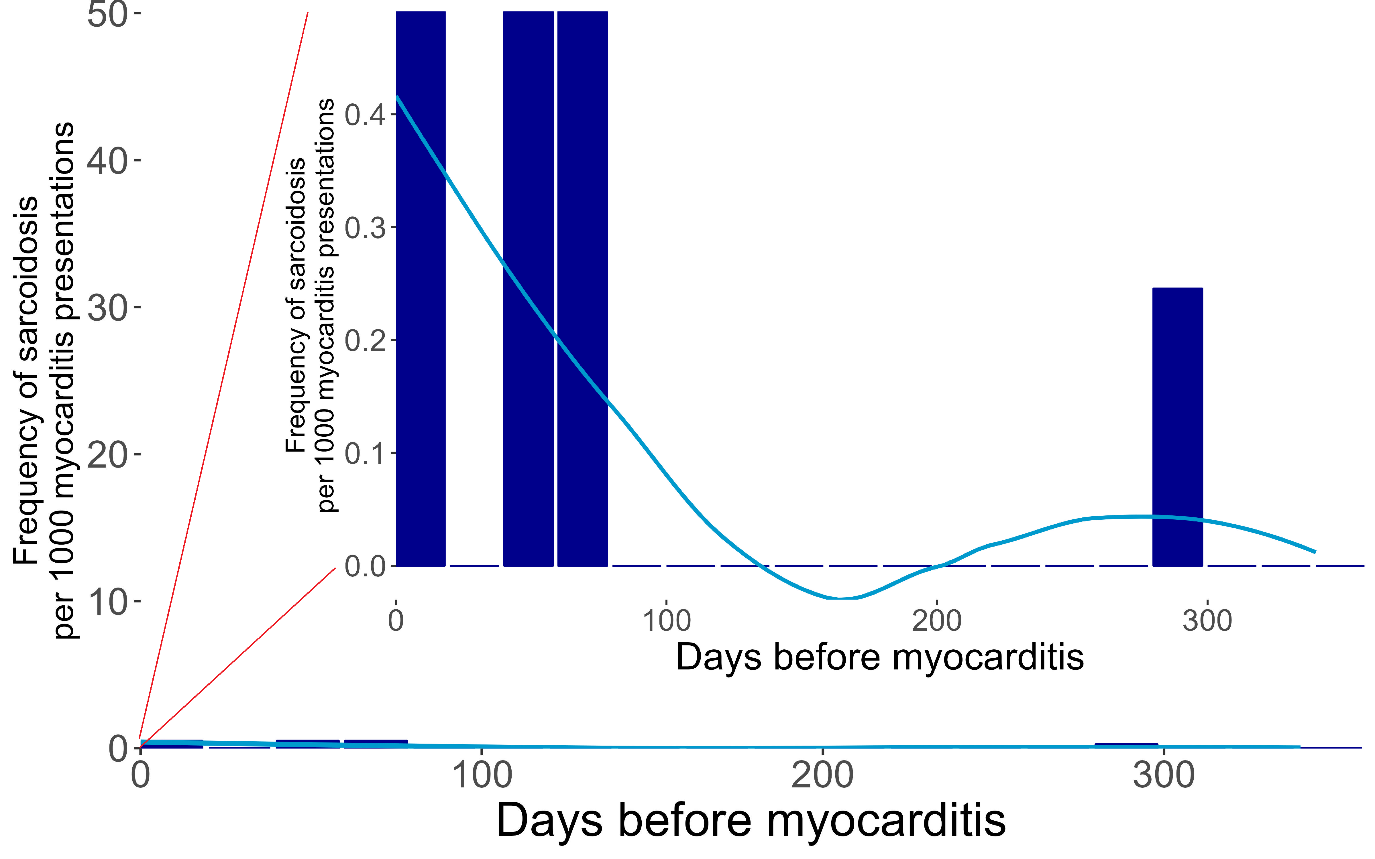


(l)

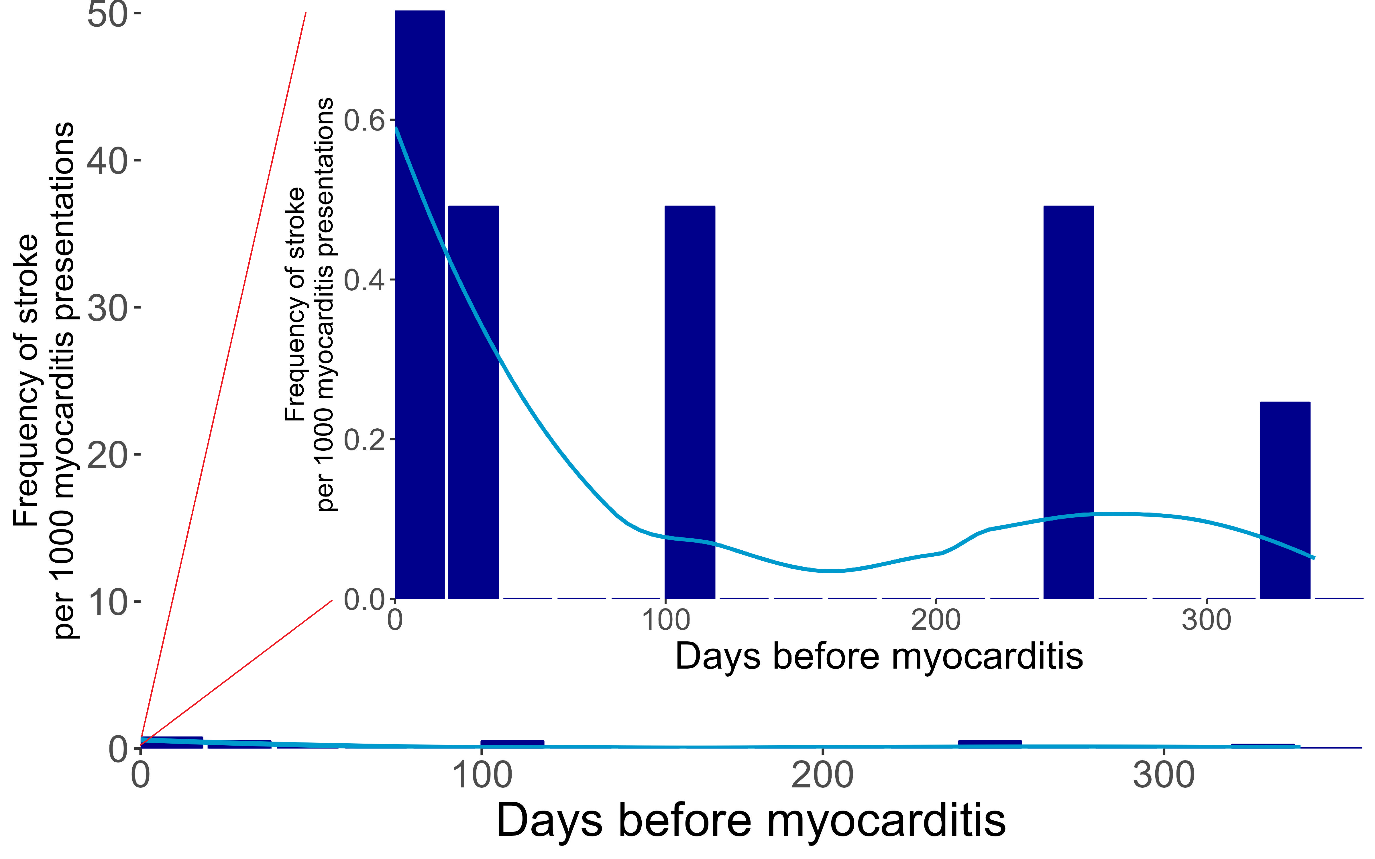


(m)

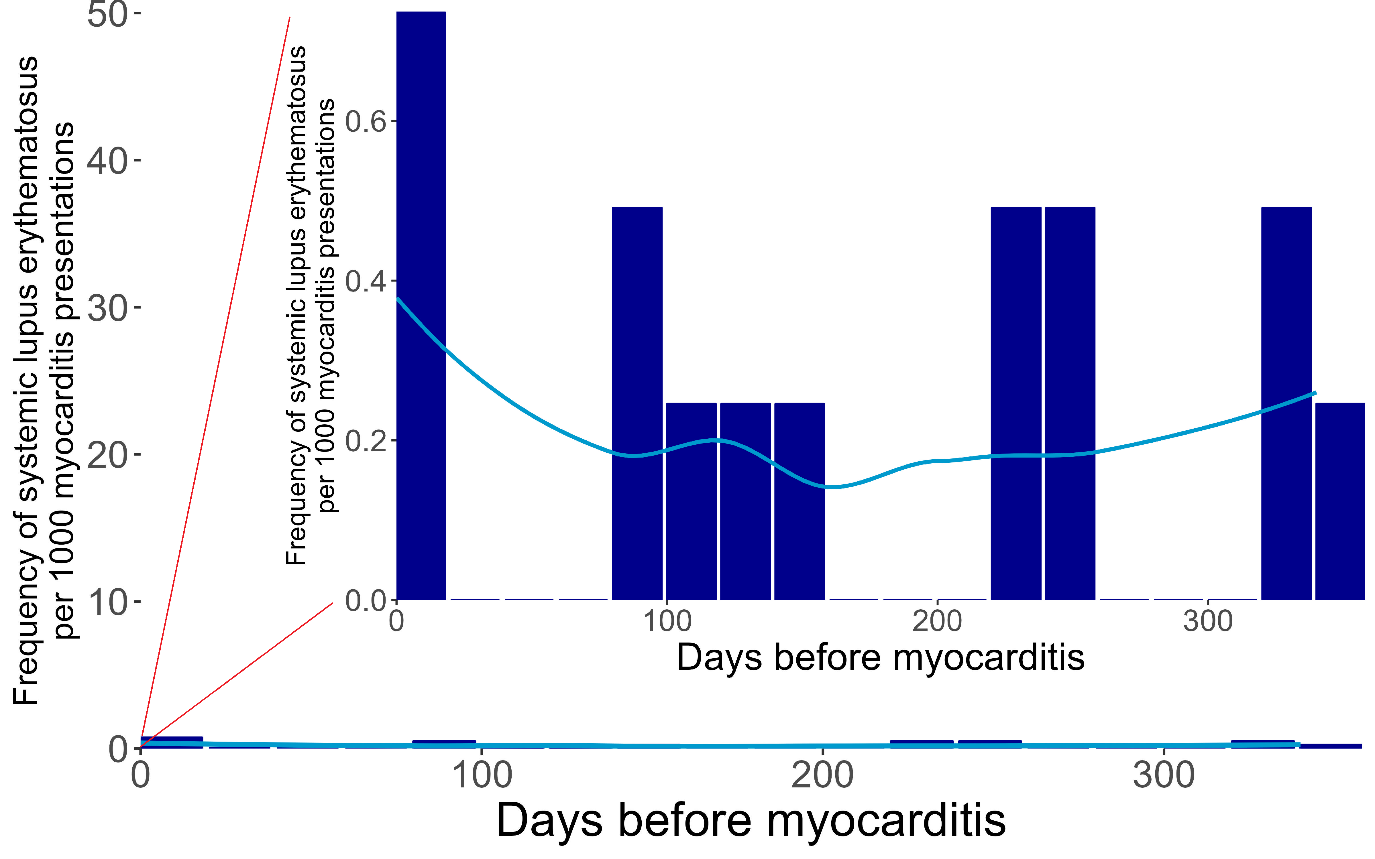


(n)

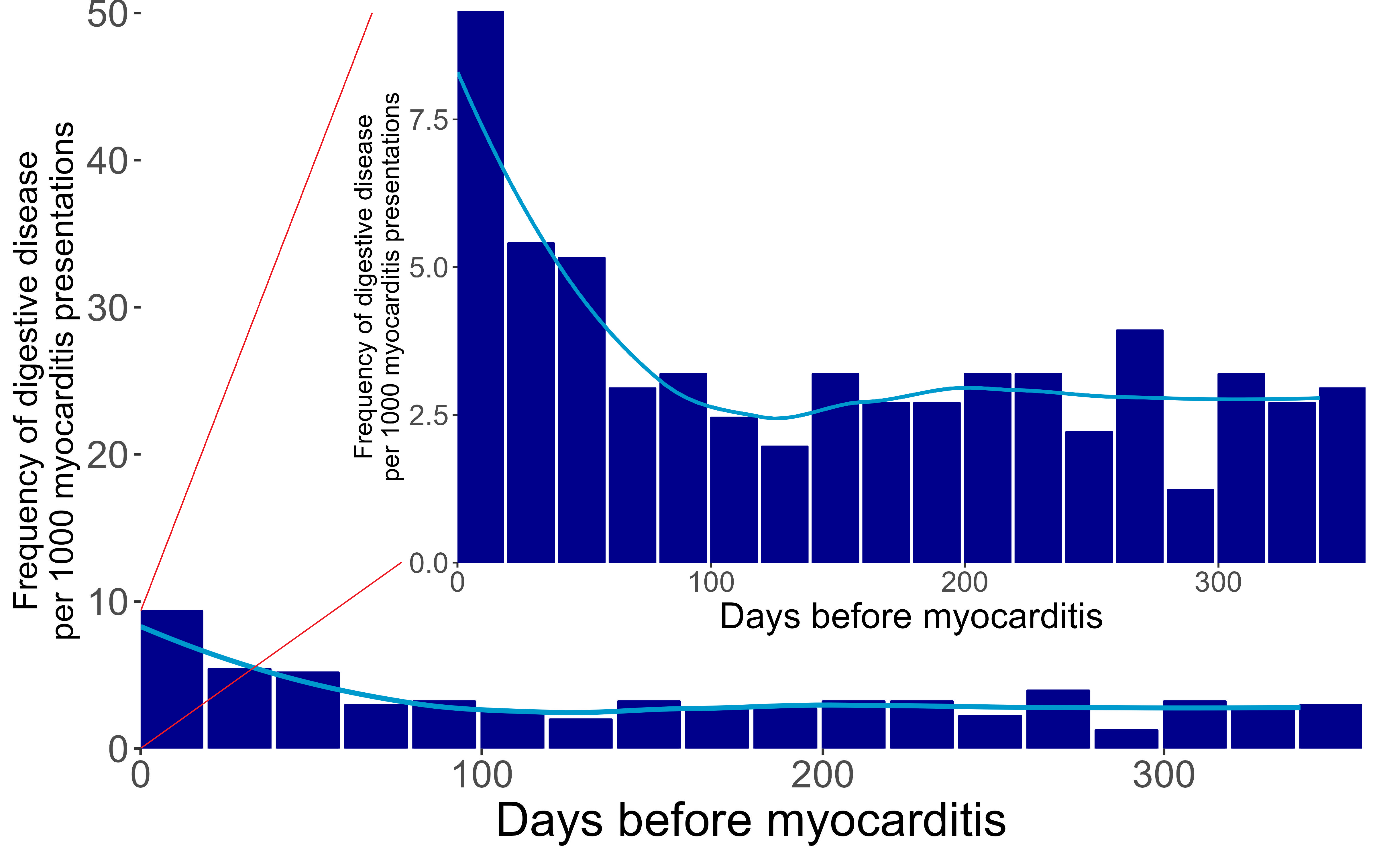


(o)

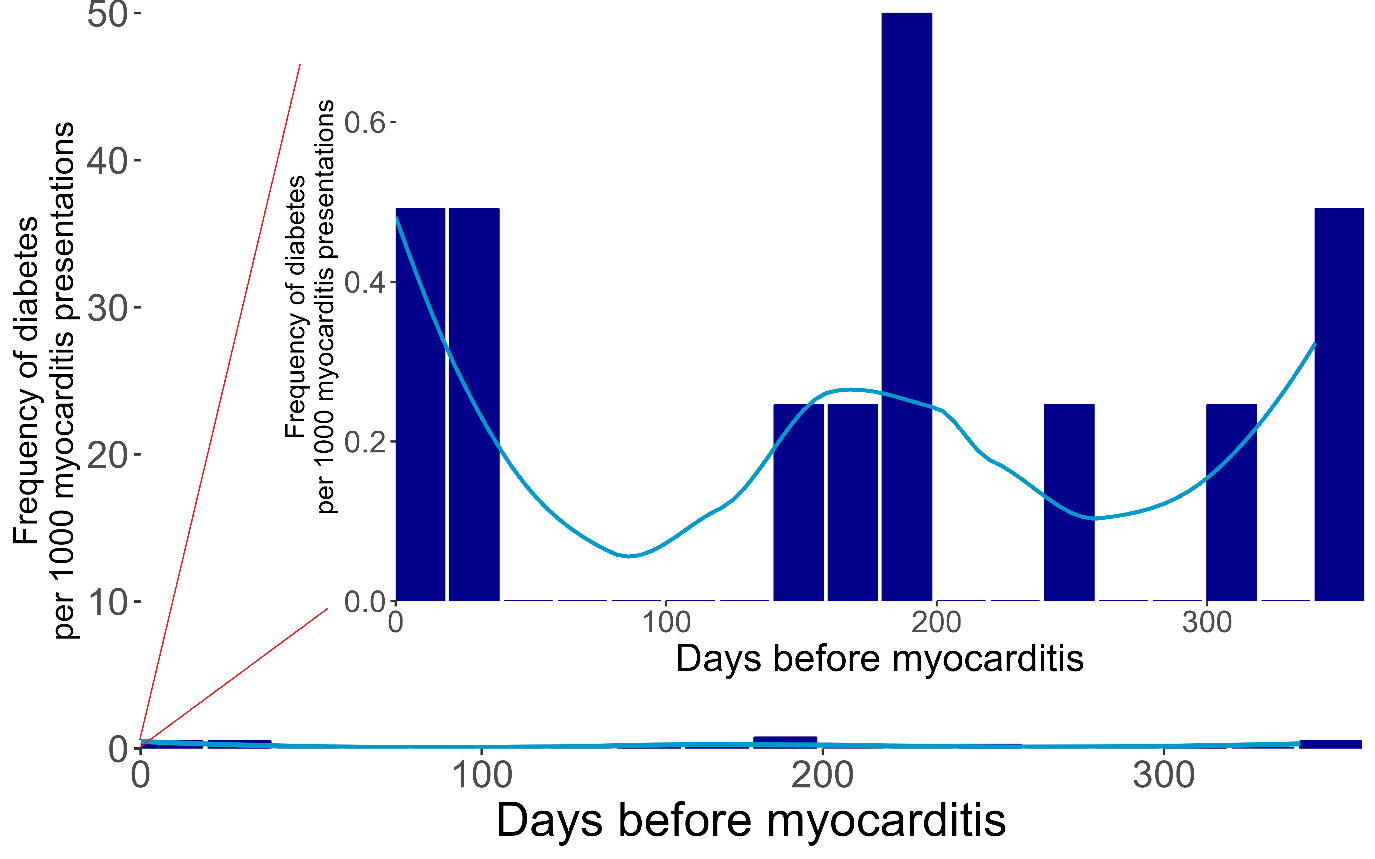


(p)
**
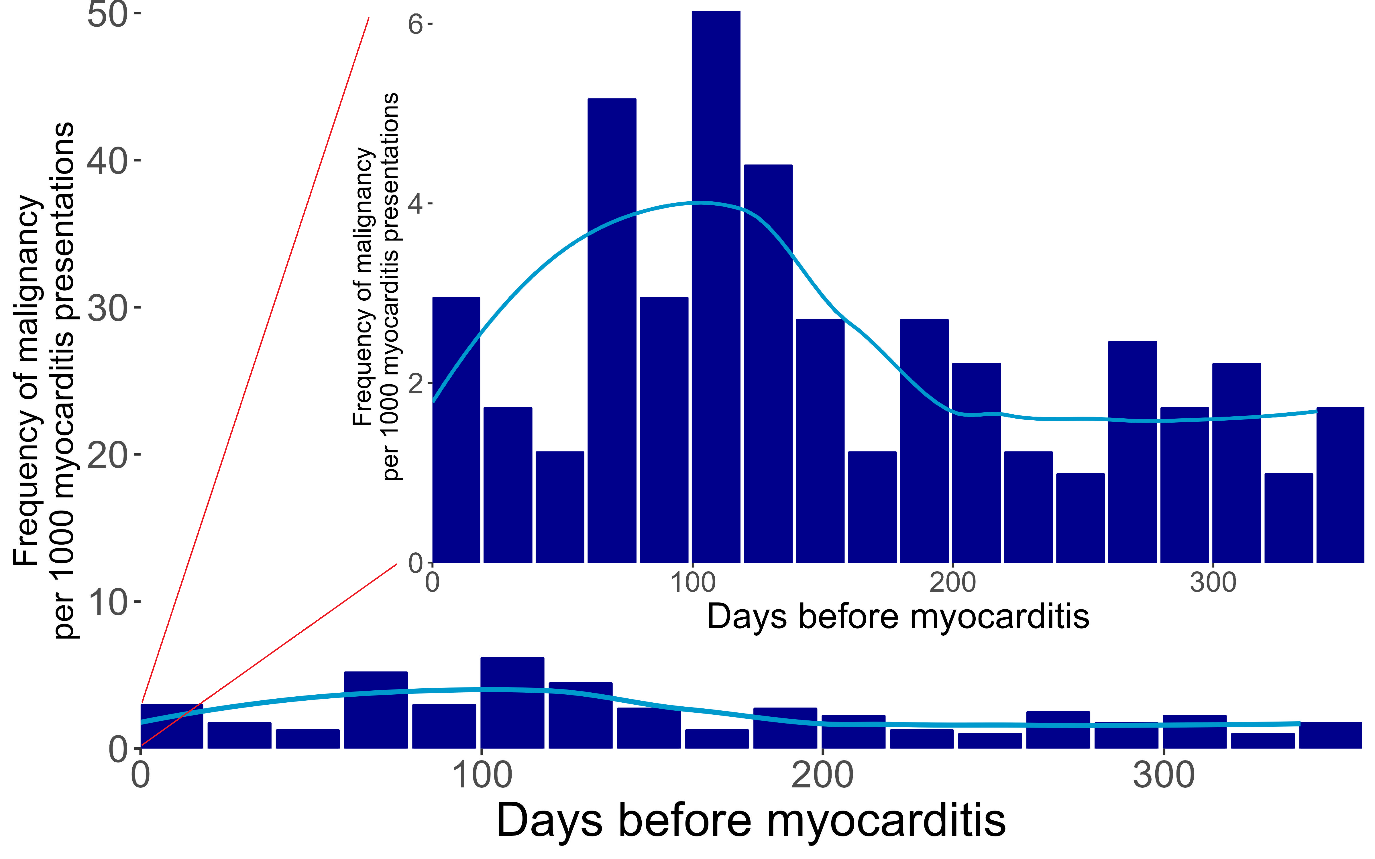
Supplementary Figure 2: frequency histogram of disease specific admission according to time prior to myocarditis admission**

1. **Myocardial infarction**
2. **Pericarditis**
3. **Heart failure**
4. **Influenza**
5. **Ventricular arrhythmia**
6. **Covid-19**
7. **Respiratory disease**
8. **Autoimmune disease**
9. **Atrial fibrillation**
10. **Myositis**
11. **Sarcoidosis**
12. **Stroke**
13. **Systemic lupus erythematosus**
14. **Digestive disease**
15. **Diabetes**
16. **Malignancy**

Higher frequencies immediately before myocarditis (on the left of the histogram) indicate a tight temporal association between the respective disease and myocarditis.
Diagnoses required to be the primary diagnosis for the admission.
Smoothed line generated by locally estimated scatterplot smoothing.
